# Elevation of Serum Cytokine Profiles and Liver Metabolomic Normalization in Early Convalescence of COVID-19 Patients

**DOI:** 10.3389/fmed.2021.626633

**Published:** 2021-07-07

**Authors:** Yan Lou, Xiaoying He, Mingxia Deng, Xingjiang Hu, Xi Yang, Lin Liu, Yunzhen Hu, Lingjuan He, Jiali Wang, Li Zhang, Qingwei Zhao, Xiaoyang Lu, Yunqing Qiu

**Affiliations:** State Key Laboratory for Diagnosis and Treatment of Infectious Diseases, Key Laboratory for Drug Evaluation and Clinical Research of Zhejiang Province, Department of Clinical Pharmacy, The First Affiliated Hospital, Zhejiang University School of Medicine, Hangzhou, China

**Keywords:** SARS-CoV-2, cytokines, metabolomics, UPLC/Q-TOF-MS, liver repair

## Abstract

Coronavirus disease 2019 (COVID-19) has become a global public health concern. We aimed to study the cytokine profile during the convalescent phase and its association with liver functions. We performed a retrospective study to investigate the longitudinal dynamic serum cytokine, liver function, and metabolomic profiles, as well as their potential correlations, from the viral replication phase to early convalescence. Our results demonstrated that liver injury was common. Liver injury was significantly associated with higher levels of interleukin (IL)-6 and IL-10 (*p* < 0.05). However, alanine aminotransferase levels decreased during the first week after hospital discharge (*p* < 0.01). In parallel, T-cell and B-cell immune response-stimulating cytokine IL-4, but not IL-2, was significantly elevated (*p* < 0.05). Furthermore, interferon-γ (IFN-γ) and tumor necrosis factor-α (TFN-α) levels increased, in contrast to the decrease in IL-6 and IL-10 levels; liver function returned to normal. The metabolomic analysis supported active recovery during early convalescence of COVID-19 patients that had distinct metabolic profiles associated with the hepatic tricarboxylic acid cycle, amino acid metabolism, and lipid metabolism. In addition, we identified a metabolomic association of IL-4 with liver repair. Our findings suggest that discharged patients continue to recover from the physiological effects of COVID-19, and the association of IL-4, IL-6, and IL-10 levels with metabolic changes and liver function repair may have important implications for clinical manifestations and treatment of COVID-19.

## Introduction

Coronavirus disease 2019 (COVID-19) has rapidly spread worldwide and has become a significant public health challenge. As of June 30, 2020, over 10,000,000 cases have been confirmed, with more than 500,000 deaths in 213 countries, and the numbers continue to rise rapidly.

Numerous studies have reported that COVID-19 severity correlates with serum inflammatory cytokine concentrations (1), and mortality often results from cytokine storm (2). Uncontrolled cytokine storm has also been implicated as a central factor contributing to severe acute respiratory syndrome coronavirus (SARS-CoV), and other severe viral infections (3, 4). Several cytokines including interleukin (IL)-6, IL-10, interferon-γ (IFN-γ), and tumor necrosis factor-α (TNF-α) have been observed to increase dramatically during acute infection in COVID-19 patients (1, 5, 6). However, previous serum cytokine profiling has been focused on acute infection of SARS-CoV-2; thus, very limited information has been reported on cytokine levels and kinetics during the convalescent phase of COVID-19.

Liver enzyme abnormality has also been observed in COVID-19 patients, and it appears to be correlated with disease severity (7). Cytokine storm and direct infection of the liver have been suggested to contribute to liver injury, and in some cases, liver failure in patients with COVID-19 (8, 9). To date, no studies have reported on the repair of liver damage in convalescent patients. However, in response to helminthic infections, the type 2 immune response (10), specifically IL-4-dependent macrophage proliferation and activation, is required to promote repair of both the liver and lung.

Furthermore, there is an immense metabolic demand during liver repair and regeneration. Cytokines have been shown to mediate several metabolic changes *via* a pathway that is commonly initiated through their regulation of the immune system (11). On the other hand, metabolites are required to regulate the homeostasis of cellular activities in hosts (12), such as resolving inflammation from viral infections (13). Metabolomics of H1N1 influenza virus-infected murine lungs identified that metabolic pathways and association clusters were related to inflammatory cytokines (14). Metabolomics has also been successfully used to identify severe drug-induced liver injury, as well as answer important biological questions (15–17).

Therefore, we have performed a retrospective study designed to investigate the longitudinal dynamic serum cytokine profile, liver functions, and metabolomic profiles in infected patients from the viral replication phase through to convalescence.

## Materials and Methods

### Patients

From January 19 to March 29, 2020, 102 SARS-CoV-2-infection-diagnosed patients were admitted to the First Affiliated Hospital, Zhejiang University School of Medicine. All patients were confirmed to be SARS-CoV-2 nucleic acid positive by real-time fluorescent RT-PCR. Patients were diagnosed in accordance with the World Health Organization's interim guidelines for COVID-19. Data were collected at our hospital. Our study was approved by the Ethics Committee of the First Affiliated Hospital of Zhejiang University School of Medicine (2020 llT-7). The categorization of mild vs. severe COVID-19 patients was conducted according to COVID-19 Diagnosis and Treatment Guideline (Trial 5th version) (Supplementary Material).

### Blood Sampling

Venous whole blood samples were collected from patients during early morning, prior to breakfast. All samples were immediately shipped to the biosafety level 3 laboratory in our hospital. The convalescent blood samples were collected from discharged patients who had met the official hospital discharge criteria, specifically a negative result from two consecutive COVID-19 nucleic acid tests and the disappearance of major clinical signs.

### Serum Cytokine Measurement

Serum was separated and stored at −80°C for cytokine detection. The IL-2/IL-4/IL-6/IL-10/TNF-α/IFN-γ cytokine assay kits (Cat No#: 8930960) were provided by Agilent Biosciences (Agilent Technologies, California, USA). The cytokines were detected using flow cytometry (ACEA NovoCyte, Agilent Technologies, California, USA) and analyzed using Novocyte kit software, according to the manufacturer's instructions.

### Metabolomic Analysis

A total of 123 blood samples were included in our final analysis, covering both the viral replication (72 samples) and convalescent (41 samples) phases, and 10 plasma samples were collected from healthy participants to serve as controls. The collected samples were centrifuged at 3,000 rpm for 5 min and the supernatant was stored at −80°C before analysis. Metabolites were extracted from plasma, and UPLC-MS/MS analysis was performed using a Waters Acquity UPLC coupled with a Xevo G2-Q-Tof (Waters, Milford, MA, USA) in both positive and negative modes. The obtained raw data were pre-processed using Progenesis QI ver. 2.2 (Nonlinear Dynamic). Metabolites were identified by searching the HMDB library (https://hmdb.ca/spectra/ms/search). Pathway analysis was performed using the MetaboAnalyst 4.0 online tool (http://www.metaboanalyst.ca/). Detailed information is outlined in supporting documents.

### Statistical Analyses

Statistical analyses were performed using the Statistical Package for the Social Sciences (SPSS, v. 18.0; SPSS, Inc., Chicago, IL, USA). All tests were two-tailed, and *p*-values < 0.05 were considered indicative of statistical significance. Student's *t*-test or the nonparametric Mann–Whitney *U*-test, as appropriate, was used for comparisons of continuous data. Categorical data were compared by Fisher's exact test and correlation analysis was calculated by Pearson correlation or Spearman correlation test.

## Results

### Demographic and Clinical Characteristics of COVID-19 Patients

**Figure 1:**
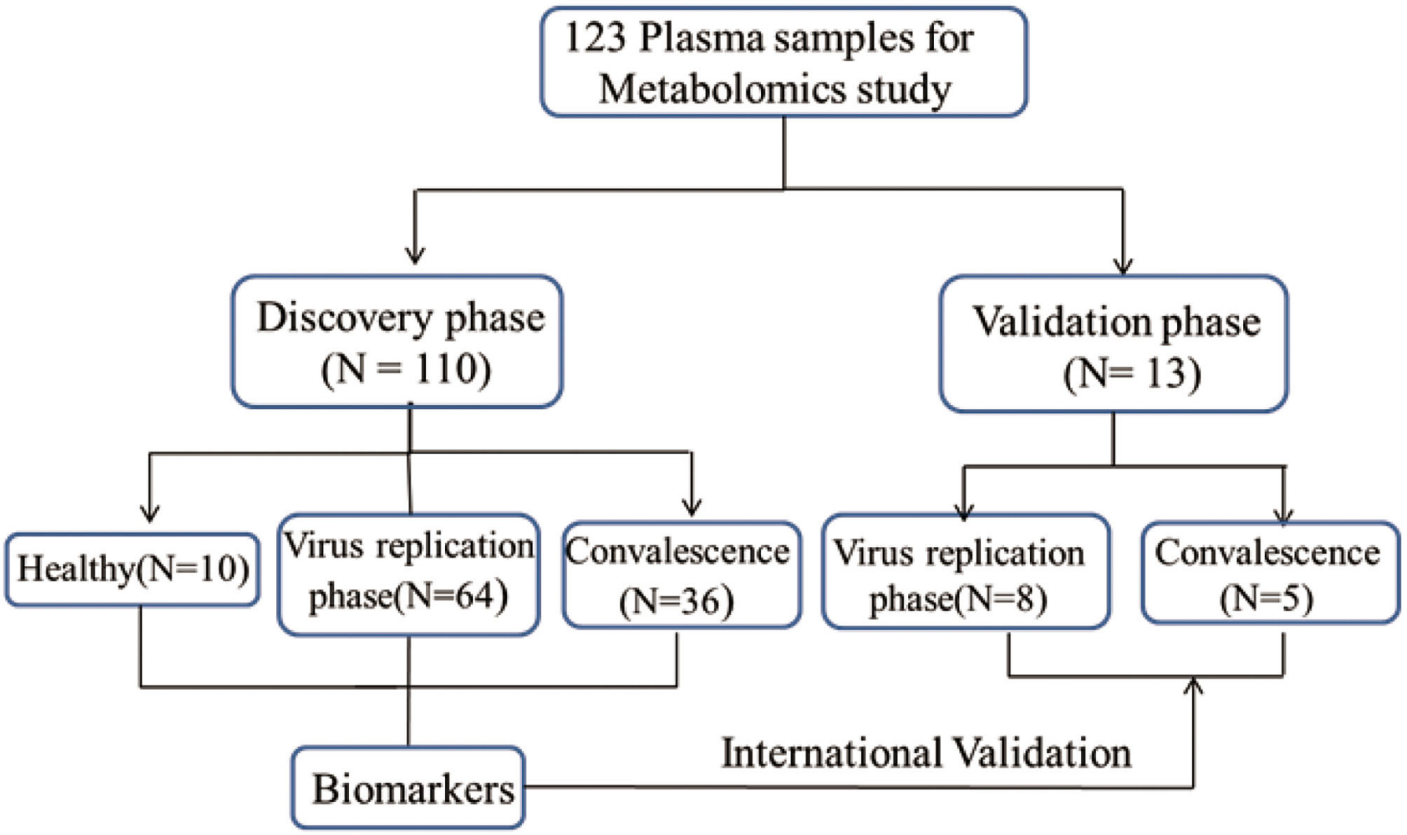
Design of the metabolomics study. This study, involving 123 plasma samples, included both discovery group and validation group. Through the analysis of the data from the discovery set, we screened and identified metabolites that were closely related to COVID-19. To validate the reliability of results from discovery set, an independent set of 13 plasma samples was collected and all samples were correctly identified by a random forest machine learning model based on the identified metabolites (Supplementary Table 9).

We conducted a retrospective study of all patients (102) with confirmed SARS-CoV-2 infection in our hospital. Two patients were subsequently excluded from the final analysis because they were discharged from the hospital the next day. The remaining 100 patients were included in the analysis. In total, 123 blood samples was involved in metabolomics analysis including both discovery group (*N* = 110) and validation group (*N* = 13) (Figure 1). The median age of the patients was 54.28 years. Most of the infected patients were men (61; 61%); less than half had underlying diseases (48; 48%), including diabetes (15; 15%), hypertension (35; 35%), cardiovascular disease (8; 8%), pulmonary disease (4; 4%), fatty liver (4; 4%), chronic kidney disease (5; 5%), and HBV (4; 4%). On admission, most patients had fever (83; 83%), cough (69; 69%), and other common symptoms including phlegm (40; 40%), chest distress (25; 25%), myalgia (18; 18%), and fatigue (14; 14%). There were only a small number of patients presenting with headache (9; 9%), dizziness (7; 7%), diarrhea (8; 8%), and nausea or vomiting (7; 7%) (Table 1).

**Table 1:** Demographics and baseline characteristics of patients infected with COVID-19.

**Variables**	**All patients**	**Liver function abnormality**	**Liver function normality**	***P***
	**(*N* = 100)**	**(*N* = 62)**	**(*N* = 38)**	
**Demographics**				
Age (years), mean ± SD	54.28 ± 16.00	55.73 ± 16.74	51.92 ± 14.62	0.250
Male sex	61 (61.00)	38 (61.29)	23 (60.53)	0.939
**Chronic medical illness**				
Diabetes	15 (15.00)	11 (17.74)	4 (10.53)	0.327
Hypertension	35 (35.00)	23 (37.10)	12 (31.58)	0.574
Cardiovascular disease	8 (8.00)	4 (6.45)	4 (10.53)	0.474
Pulmonary disease	4 (4.00)	3 (4.84)	1 (2.62)	1.000
Fatty liver	4 (4.00)	4 (6.45)	0	0.294
Chronic kidney disease	5 (5.00)	3 (4.84)	2 (5.26)	1.000
HBV	4 (4.00)	3 (4.84)	1 (2.62)	1.000
**Symptoms**				
Fever	83 (83.00)	52 (83.87)	31 (81.58)	0.767
Cough	69 (69.00)	44 (70.97)	25 (65.79)	0.587
Phlegm	40 (40.00)	28 (45.16)	12 (31.58)	0.178
Chest distress	25 (25.00)	19 (30.65)	6 (15.79)	0.096
Dizziness	7 (7.00)	4 (6.45)	3 (7.89)	1.000
Myalgia	18 (18.00)	9 (14.52)	9 (23.68)	0.247
Headache	9 (9.00)	6 (9.68)	3 (7.89)	1.000
Diarrhea	8 (8.00)	6 (9.68)	2 (5.26)	0.707
Nausea or vomiting	7 (7.00)	5 (8.06)	2 (5.26)	0.706
Fatigue	14 (14.00)	12 (19.35)	2 (5.26)	0.049
**Laboratory parameters (mean** **±** **SD)**				
Leucocytes (× 10^9^/L)	7.44 ± 4.63	7.82 ± 4.92	6.82 ± 4.11	0.295
Neutrophils (%)	66.93 ± 28.07	68.05 ± 29.36	65.09 ± 26.08	0.153
Lymphocytes (× 10^9^/L)	1.29 ± 2.64	1.48 ± 3.33	0.98 ± 0.45	0.301
Hemoglobin (g/L)	134.60 ± 16.69	135.56 ± 17.26	133.03 ± 15.81	0.463
Platelets (× 10^9^/L)	202.40 ± 83.57	188.89 ± 70.71	224.45 ± 98.18	0.127
Total bilirubin (μmol/L)	12.98 ± 9.80	14.82 ± 11.65	9.97 ± 4.29	0.040
Direct bilirubin (μmol/L)	6.34 ± 6.31	7.39 ± 7.67	4.63 ± 2.18	0.027
ALT (μmol/L)	29.55 ± 33.70	35.32 ± 40.97	20.13 ± 11.17	0.005
AST (μmol/L)	28.10 ± 17.89	32.77 ± 20.77	20.47 ± 7.01	<0.001
ALP (U/L)	76.06 ± 69.30	79.59 ± 5.29	70.26 ± 34.14	0.867
Albumin (g/L)	38.44 ± 5.68	37.59 ± 5.29	39.83 ± 6.08	0.055
Serum creatinine (μmol/L)	87.50 ± 96.72	80.65 ± 38.98	98.68 ± 149.38	0.887
INR	1.10 ± 1.14	0.99 ± 0.07	1.29 ± 1.82	0.496
D-dimer (ng/ml)	1049.34 ± 4,406.81	1384.48 ± 5,577.97	502.53 ± 412.87	0.204
**Infection-related biomarkers**				
Procalcitonin (ng/ml)	0.12 ± 0.24	0.16 ± 0.29	0.06 ± 0.10	<0.001
Erythrocyte sedimentation rate (mm/h)	43.76 ± 27.95	45.42 ± 25.82	41.05 ± 31.30	0.323
Serum ferritin (ng/ml)	606.49 ± 754.97	641.64 ± 745.06	549.15 ± 777.46	0.478
C-reactive protein (mg/L)	32.00 ± 35.94	33.95 ± 33.44	28.81 ± 39.95	0.053

*Data expressed as n (%) and mean ± SD. HBV, hepatitis B virus; ALP, alkaline phosphatase; AST, aspartate aminotransferase; ALT, alanine aminotransferase; INR, International normalized ratio*.

### Liver Injury and Recovery in COVID-19 Patients

**Figure 2:**
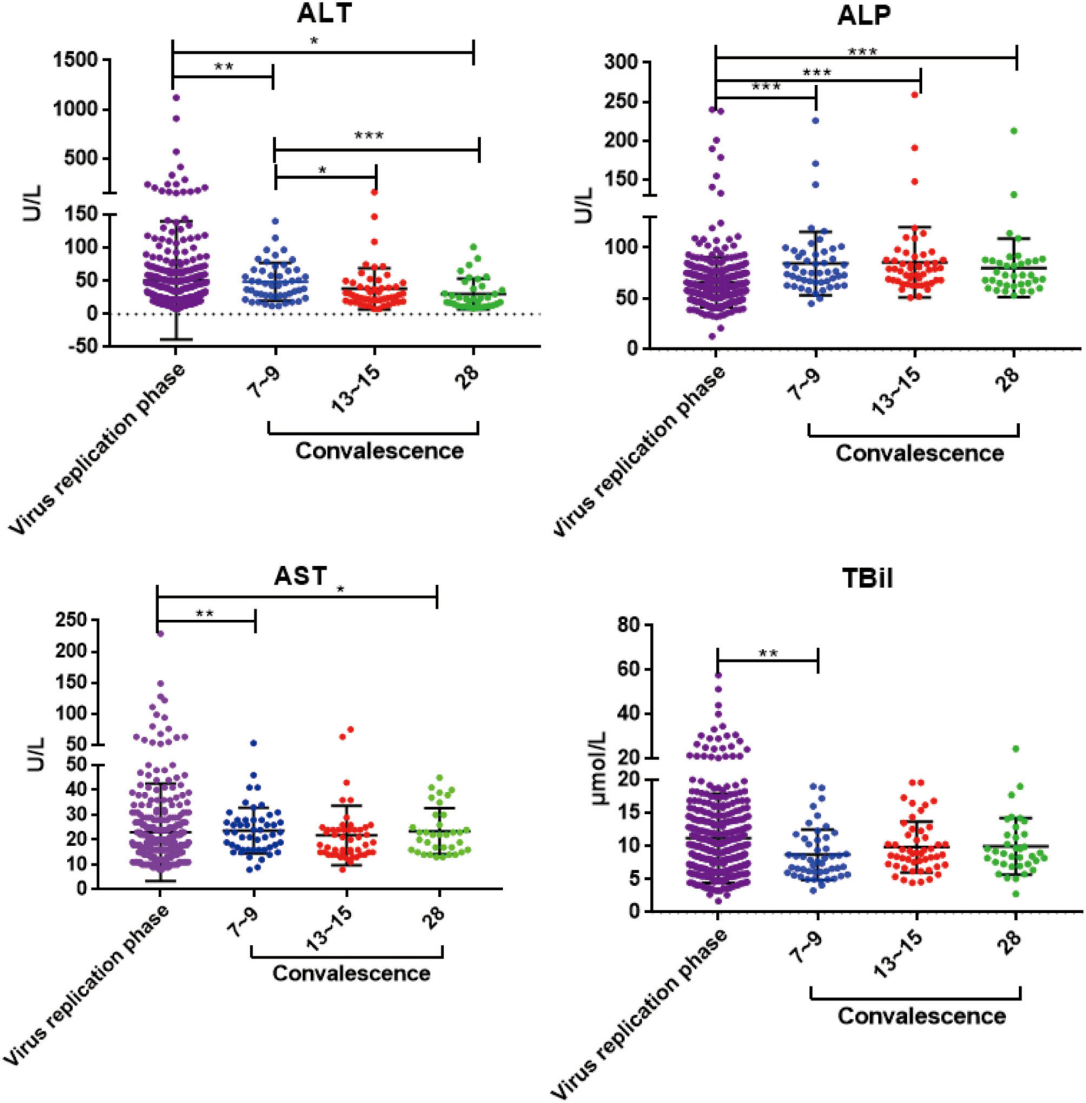
The dynamic changes of ALT, AST, ALP, and TBiL in virus replication phase and early convalescence. Levels of significance: *p*-value: ***, <0.001; **, <0.01; *, <0.05.

To investigate whether liver enzyme abnormality occurred in this COVID-19 patient cohort, multiple liver function indicators, including serum alanine aminotransferase (ALT), aspartate aminotransferase (AST), alkaline phosphatase (ALP), and total bilirubin (TBiL), were collected from the onset of the disease. Among the 100 COVID-19 patients in our hospital, 62 (62%) had increased levels of ALT, AST, ALP, and TBiL (Table 1 and Supplementary Table 3). These results suggest that liver injury is a common clinical feature of SARS-CoV-2 infection. To determine the course of liver recovery, liver function indicators were measured weekly after patients were discharged from hospital. Serum ALT levels decreased within the first week after discharge (*p* < 0.01) (Figure 2). Levels of ALT continued to decline significantly through the second (*p* < 0.05) and fourth weeks (*p* < 0.001) (Figure 2). Significant changes to AST, ALP, and TBiL levels also occurred toward the normal range (Figure 2), indicating the active recovery of liver functions during early convalescence of COVID-19.

### Association of Elevated IL-6 and IL-10 With Liver Injury

**Figure 3:**
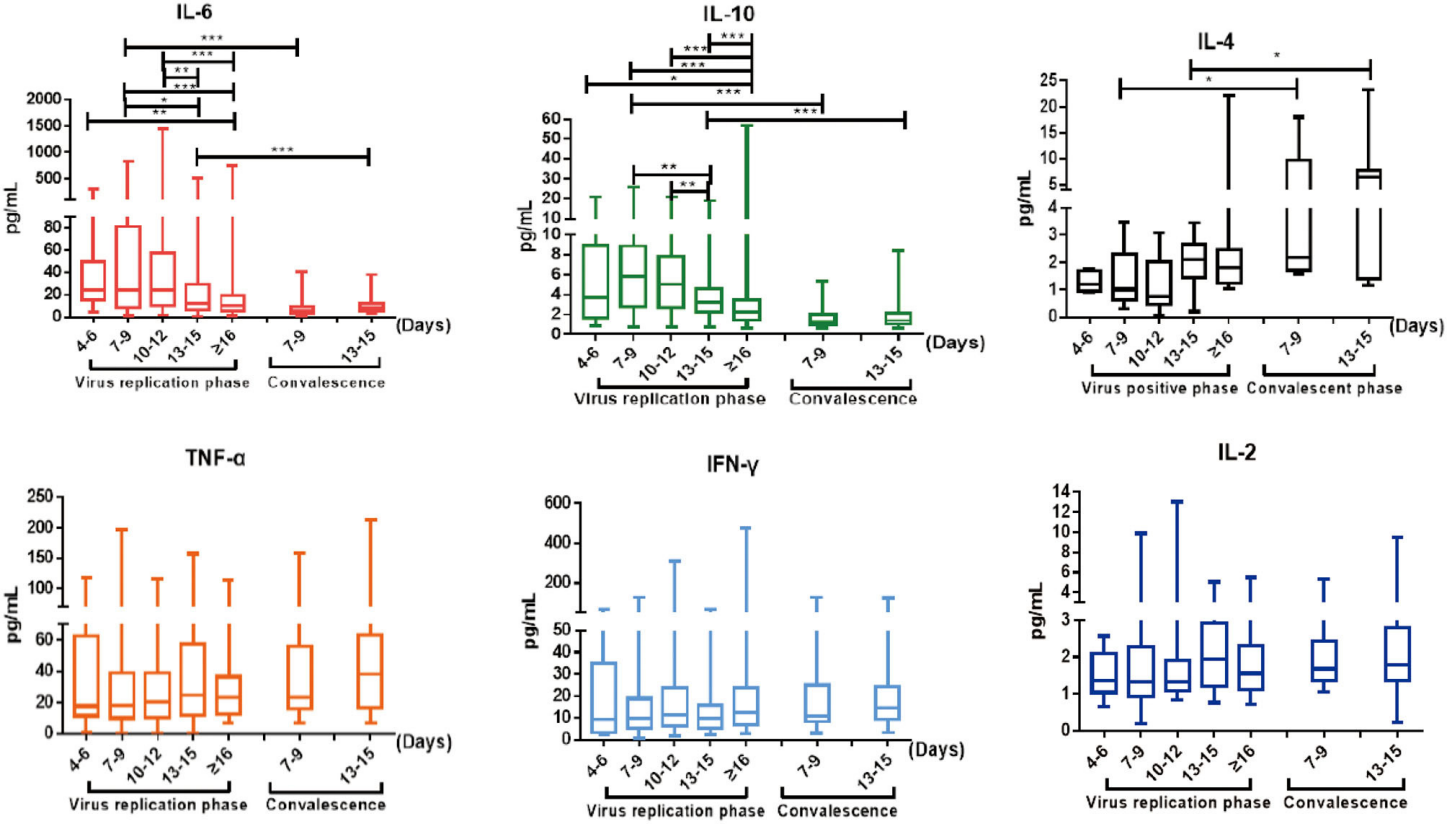
The dynamic changes of IL-6, IL-10, IL-4, TNF-α, IFN-γ, and IL-2 in virus replication phase and early convalescence. Levels of significance: *p*-value: ***, <0.001; **, <0.01; *, <0.05.

Cytokine analysis revealed that cytokines IL-6 and IL-10 were elevated in COVID-19 patients on admission and continued to increase significantly as the disease progressed (Figure 3), consistent with previous reports (1). IL-6 and IL-10 levels gradually declined into normal ranges as the viral RNA became undetectable (*p* < 0.05) (Figure 3). Furthermore, as shown in Supplementary Table 5, IL-6 showed sustained higher levels in the liver impaired group, compared with the normal liver function group. Elevated levels of IL-6 became significant 7–9 days after disease onset (*p* = 0.034) and became even more pronounced as the disease progressed (*p* = 0.001 at days 13–15 and ≥16) (Supplementary Table 5). These results suggest a possible correlation between liver damage and the inflammatory responses induced by SARS-CoV-2 infection. In addition, increased levels of IL-6 and IL-10 were also significantly correlated with disease severity and patient age (Supplementary Table 5).

### Elevation of Serum IL-4 Level in Early Convalescence

Cytokine profiles in convalescent patients remain less characterized; thus, we performed serum cytokine analysis weekly after patients were discharged from hospital. Significantly, IL-4 was elevated in convalescent patients within the first week after discharge (*p* = 0.036) and continued to stay high at week 2 (*p* = 0.022) (Figure 3), even though IL-4 had no significant fluctuation through the viral replication phase as previously reported (1). IL-4 plays a key role in promoting naïve T cells to develop into Th2-like cells (18) and immunoglobulin class-switching from IgM to IgE and IgG (19). The observed elevation of IL-4 might suggest that there is a differential set of regulatory T cell and B cell immune responses occurring in early convalescence of COVID-19 patients.

Antiviral cytokines IFN-γ and TNF-α continued to stay at high levels as patients transitioned from the viral replication phase to convalescence, whereas IL-6 and IL-10 gradually declined and normalized (Figure 3). In particular, the serum levels of both IFN-γ and TNF-α showed meaningful increases above normal ranges from weeks 1 to 2 after hospital discharge, suggesting an additional boost of T cell immune responses (IFN-γ: 10.98 to 14.33 pg/ml; TNF-α: 23.27 to 37.96 pg/ml; Supplementary Table 4). In contrast, IL-2 levels remained within normal ranges throughout the course of the study (Figure 3).

### Plasma Metabolomic Alternations Associated With Viral Replication and Convalescent Phases

**Figure 4:**
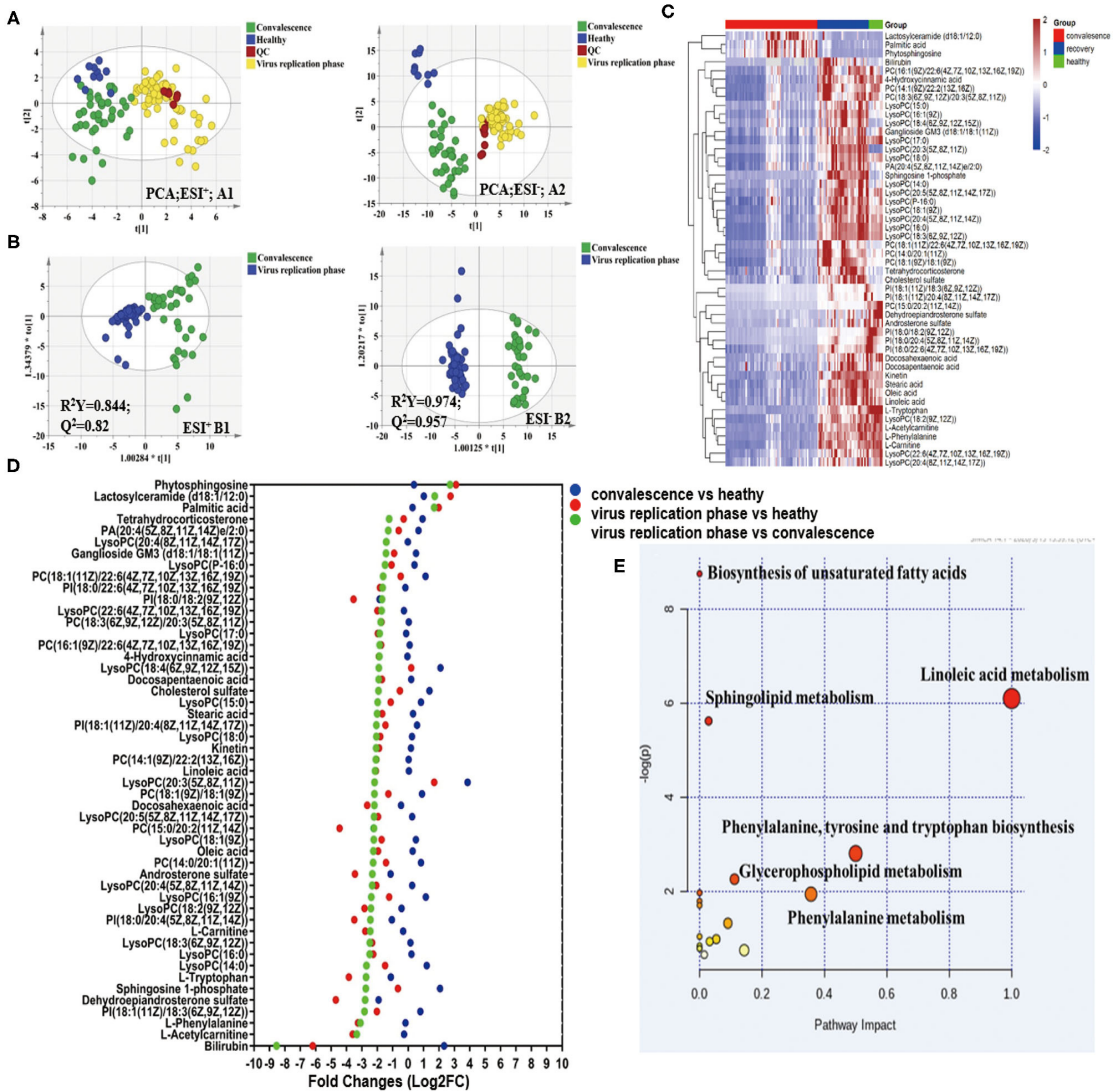
Metabolomic profiling of COVID-19 patient plasma. **(A)** Principal components analysis of the metabolites across the three study groups based on the data of ESI+ and ESI– modes. Colors display the subjects from different groups (blue, normal controls; yellow, virus replication group; green, convalescent group; and red, QC samples). **(B)** OPLS-DA plot of COVID-19 patients in virus replication phase and early convalescence. **(C)** Heatmap of 50 selected metabolites, the colors changing from red (upregulated metabolites) to blue (downregulated metabolites). **(D)** Cleveland plots. Fifty metabolites are ranked according to their fold changes in the virus replication group vs. convalescent group (the highest fold changes on the top; the lowest on the bottom). Fold changes in the virus replication group vs. normal controls and the convalescent group vs. normal controls are also shown. **(E)** MetPA. Pathway analysis was performed using MetaboAnalyst 4.0 for the 50 differential metabolites. The color and size of each circle are based on its *p*-value and pathway impact value, respectively.

Metabolites were extracted from plasma, and UPLC-QTOF MS analysis was performed for metabolomic analysis. From a total of 2,077 metabolic features detected in metabolomic analysis, 243 were selected based on the following selection criteria: absolute log2 FC > 1; *p*-value < 0.05; projection variable VIP > 1; the area of ROC curve > 0.8. Among them, in comparison with replication phase, 50 metabolites showed differential profiles in convalescent patients, mostly with upregulation back toward the normal levels (Figures 4A–C), demonstrating that there was a significant change in metabolites during recovery from COVID-19. Using the results obtained in the viral replication phase/convalescence comparison as a reference, we ranked metabolites from the highest fold change value to the lowest value and drew a Cleveland plot showing fold changes of each metabolite across the three pairwise comparisons (Figure 4D). Among the three groups, the metabolites in the viral replication phase notably decreased.

As shown in Figure 4E, pathway analysis identified changes in 18 pathways during convalescence (Supplementary Table 8). Three pathways, namely, linoleic acid metabolism, biosynthesis of unsaturated fatty acids, and sphingolipid metabolism, were significantly altered. They included 32 downregulated metabolites, such as triglycerides, decanoylcarnitine, and diglycerides, and 43 upregulated metabolites, such as sphingosine 1-phosphate and cholic acid (Supplementary Figure 7). Notably, many metabolites returned to the levels observed in healthy controls (Supplementary Figure 6). Together, the metabolomic analysis indicated that discharged patients continued to recover from the physiological impacts of COVID-19.

### Metabolomic Association of Inflammatory Cytokines With Liver Functions

**Figure 5:**
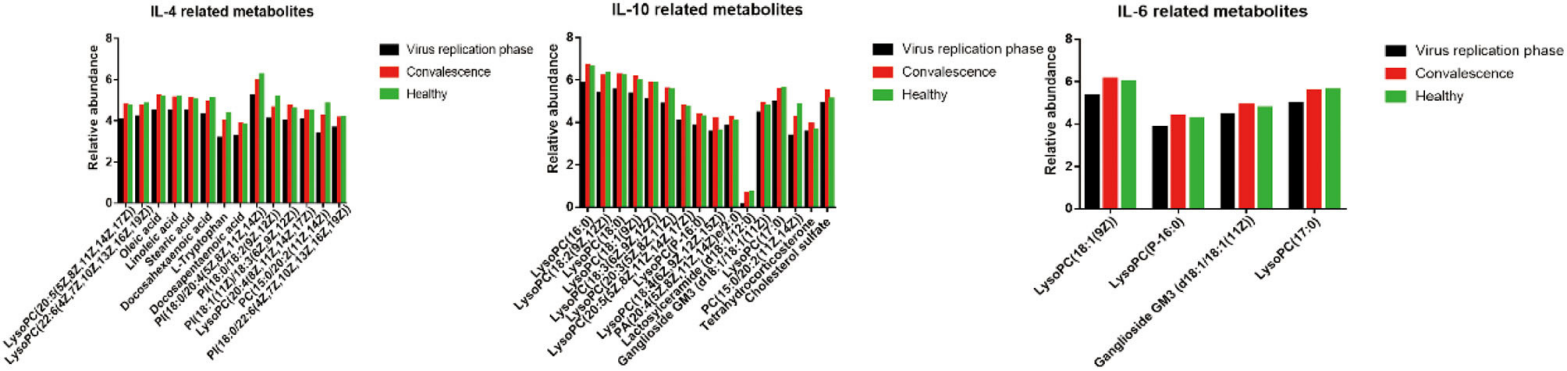
Change of expression level metabolites related to the cytokines.

Since liver injury from SARS-CoV-2 was associated with the extent of cytokine expression (Supplementary Table 6), we examined the interaction between metabolic changes of metabolites and cytokines on liver functions. As shown in Figure 5 and Supplementary Figures 4, 5, most of the upregulated metabolites in the convalescent stage were negatively correlated with IL-6, IL-10, ALT, and TBiL, while downregulated metabolites were positively correlated with IL-4. There were 28 metabolites, including amino acids, glycerophospholipids, ceramides, and unsaturated fatty acids, which were significantly correlated with cytokines (IL-4, IL-6, and IL-10) (*p* < 0.05). In addition, 10 metabolites, including amino acids, glycerophospholipids, steroids, and steroid derivatives, were significantly correlated with liver function indicators (ALT and TBiL) (*p* < 0.05) (Supplementary Figures 4, 5).

## Discussion

In this study, we aimed to analyze the liver function repair and cytokine profiles during convalescence of COVID-19, which has barely been characterized. Cytokine storm and direct infection of the liver has been suggested to contribute to liver injury, and in some cases, liver failure in patients with COVID-19 (8, 9). Our results supported the notion that liver injury is a common clinical feature of SARS-CoV-2 infection, and we observed the active recovery of liver functions during the early convalescent stage of COVID-19. Importantly, serum ALT level decreased within the first week after discharge (*p* < 0.01) and continued to significantly decline through the second (*p* < 0.05) and fourth weeks (*p* < 0.001) (Figure 2). Significant shifts of AST, ALP, and TBiL levels toward the normal range also occurred (Figure 2), indicating the active recovery of liver functions during early convalescence.

Pro-inflammatory cytokines, such as TNF-α and IL-6, are primarily involved in the promotion of inflammatory processes and have an important role in liver injury (20). Cytokine profile analysis confirmed that both IL-6 and IL-10 became significantly elevated (*p* < 0.05) during the early viral replication phase and then gradually declined into the normal range as viral RNA became undetectable (Figure 3). IL-6 and IL-10 levels were higher in patients with abnormal liver function than in those with normal liver function (Supplementary Table 5). Furthermore, after discharge from hospital, liver function normalized during the first 2 weeks of convalescence, and both inflammation-regulating cytokines continued to decline significantly (*p* < 0.05), indicating clinical improvement.

We found that immune response-stimulating cytokine IL-4, but not IL-2, was significantly elevated during the first 2 weeks of convalescence. This was a surprising observation, as no significant fluctuation of IL-4 was seen during the viral replication phase (1), which was also confirmed in our study. IL-4 is known to regulate a variety of immune responses, including differentiation of naïve T cell into Th2 cells, and immunoglobulin class switching to IgG1 and IgE in B cells. For example, IL-4 could signal through IL-4Rα to trigger specialized macrophage activation, promoting the mitigation of helminthic infection and tissue repair in the liver and lung (21), or reduce the production of C-reactive protein (CRP) by human primary hepatocytes (22). Furthermore, IL-4 polymorphism has also been associated with an increased risk of liver disease (23) and severe respiratory syncytial virus (RSV) infection (24). All things considered, it is reasonable to postulate that an increase of IL-4 levels may play an important role in the convalescence of COVID-19 through either T cells, B cells, or other type 2 immunity-associated cells, such as macrophages.

Furthermore, abnormal liver function became normalized during the first 2 weeks of convalescence (Figure 2). Additionally, the antiviral cytokines IFN-γ and TNF-α, in contrast to the decline of IL-6 and IL-10, were observed to significantly increase during convalescence of those patients (Figure 3 and Supplementary Table 4). Both IL-4 and IFN-γ were shown to downregulate the expression of SARS coronavirus receptor angiotensin-converting enzyme 2 (ACE2), in order to inhibit viral infection (25). Together, these clinical features further support the notion that type 2 immunity may contribute to liver and lung repair following injury by SARS-CoV-2. Finally, IL-13 has already been shown to share many biological functions with IL-4 and can inhibit IL-6 production through peripheral blood mononuclear cells (26). Therefore, it is recommended to include IL-13 in future cytokine profile analysis (27).

**Figure 6:**
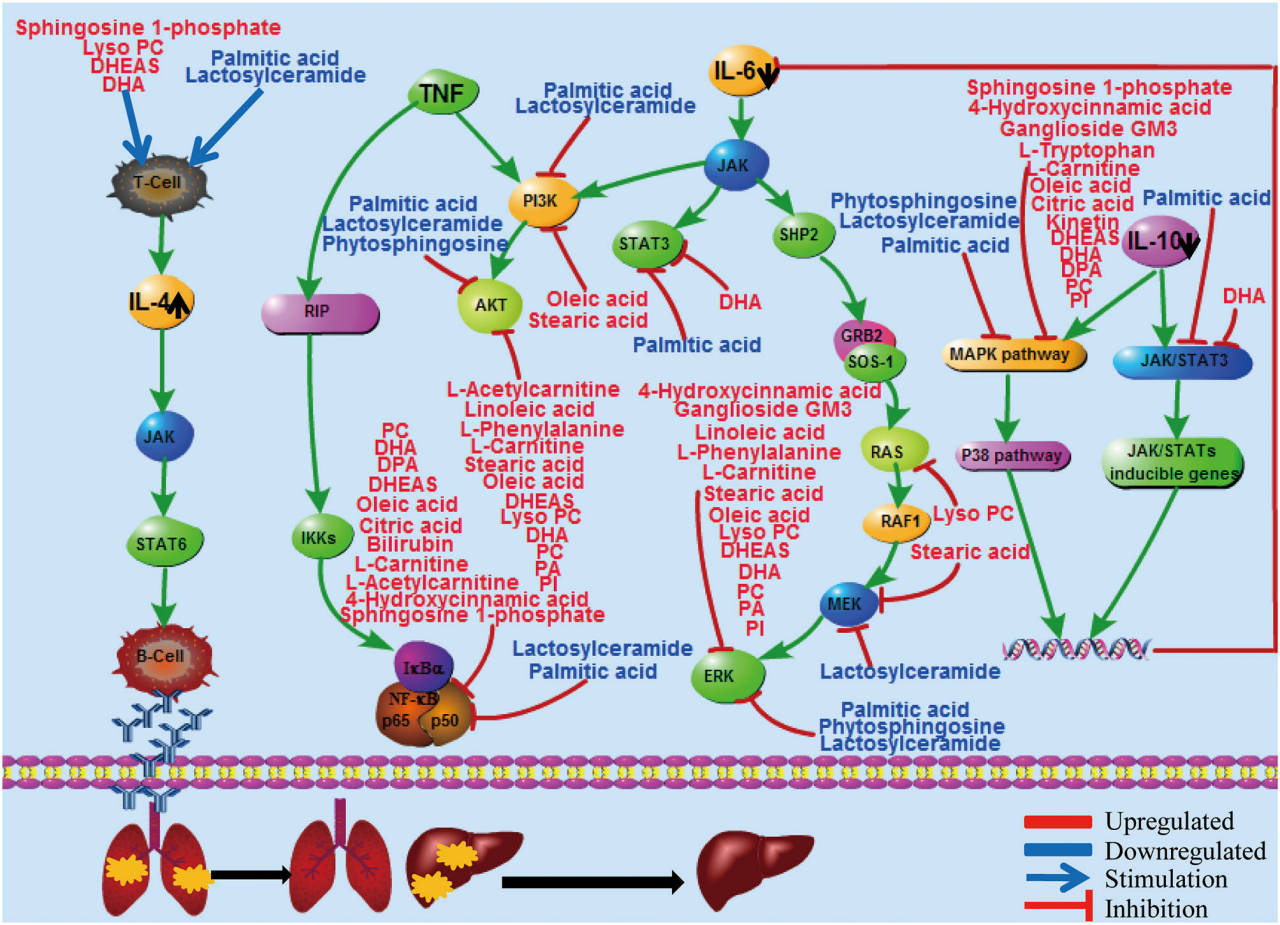
Diagram to illustrate potential relationships among liver injury repair, inflammatory cytokine reactions, and metabolic changes in early convalescence. The red color represents upregulated metabolites and blue represents downregulated metabolites. The black arrow represents the changes of cytokines in early convalescence compared to virus replication phase. Lyso PC, lysophosphatidylcholines; PC, phosphatidylcholine; DHEAS, dehydroepiandrosterone sulfate; DHA, docosahexaenoic acid; DPA, docosapentaenoic acid; PA, phosphatidic acid; PI: phosphatidylinositol.

To further corroborate the association of liver injury and repair with cytokine profile changes, metabolic changes were determined in comparison with healthy controls. Metabolomic analysis shows significantly distinct profiles of metabolites and cytokines between the viral replication and the convalescent phases, including the liver-associated amino acid, TCA cycle, steroid hormone biosynthesis, and lipid metabolism (Figure 6). Remarkably, all of these metabolic changes appear to support liver repair. For example, during convalescence, the saturated fatty acid palmitic acid decreased, potentially mitigating the apoptosis of hepatocytes (28). In contrast, the unsaturated fatty acids docosapentaenoic acid (DPA) and docosahexaenoic acid (DHA) increased, possibly promoting liver repair by inhibiting ALT (29). An increase of tryptophan may reverse liver injury by maintaining protein synthesis activity (30). Importantly, during the preparation of our manuscript, part of the metabolomic profile we identified in the viral replication phase was also observed in other studies on SARS-CoV-2 (31, 32). These results are consistent with the general notion that cellular metabolites play key roles in resolving inflammation resulting from various viral infections, including H1N1 influenza (13, 14). Together, these metabolic changes strongly indicate that the liver is undergoing active recovery.

The immune system is particularly sensitive to metabolite availability (21). On the other hand, cytokines have been shown to mediate several metabolic changes *via* a pathway that is commonly initiated through their regulation of the immune system (11). Correlation analysis identified that many metabolites were associated with proinflammatory IL-6 and anti-inflammatory IL-10 cytokine levels, suggesting a potential cytokine-mediated metabolic dysfunction from COVID-19 pathogenesis (Figure 6). IL-6 can signal through three main pathways: JAK-STAT3, SHP-2-MAPK, and PI3K-AKT. At least 14 upregulated metabolites, such as oleic acid and DHA, and 3 downregulated metabolites, such as palmitic acid and lactosylceramide, were revealed, each of which are known to inhibit these three pathways (Figure 6 and Supplementary Material). IL-10 is a major anti-inflammatory cytokine secreted by macrophages, and it exerts its effects *via* the JAK-STAT3 pathway (33). Palmitic acid and DHA levels were downregulated and upregulated, respectively, both of which may inhibit STAT3 signaling to promote liver repair. IL-4 is one of the best-known anti-inflammatory cytokines, mediating its biological roles predominantly *via* the JAK-STAT6 pathway (34). Our metabolic analysis identified four upregulated metabolites, including sphingosine 1-phosphate and DHA, and two downregulated metabolites, palmitic acid and lactosylceramide, which could stimulate T cells to produce IL-4 in early convalescence (Figure 6). The diagram in Figure 6 summarizes the potential interactions among liver injury repair, inflammatory cytokine reactions, and metabolic changes.

## Conclusion

In conclusion, by analyzing liver function repair and cytokine profiles in early convalescence of COVID-19, in tandem with the viral replication phase, we have identified that liver injury is a common clinical feature in COVID-19 patients, and it is associated with the increase of cytokine IL-6 and IL-10 levels. Importantly, serum levels of IL-4 were significantly elevated during early convalescence, suggesting a potentially important role of Th2 immune response in liver injury repair. The correlation of liver injury and repair with cytokines was further corroborated by metabolomic analysis, which identified a series of related biomarkers for the recovery of COVID-19 patients. Collectively, our new findings may have important implications in analysis of clinical manifestations and the potential therapeutic treatment of COVID-19.

## Data Availability Statement

The original contributions presented in the study are included in the article/Supplementary Material, further inquiries can be directed to the corresponding author/s.

## Ethics Statement

The studies involving human participants were reviewed and approved by the Ethics Committee of the First Affiliated Hospital of Zhejiang University School of Medicine (2020 llT-7). The patients/participants provided their written informed consent to participate in this study.

## Author Contributions

YL wrote the manuscript and designed the overall scheme. XHe analyzed the medical record information of these patients. MD and LL were responsible for the collection and interpretation of data. XHu, XY, and LH were mainly responsible for the metabolomic analysis by UPLC-MS/MS. YH and QZ participated in cytokine measurement and analysis. JW and LZ were responsible for the collection of the blood samples and shipped the samples to the biosafety level 3 laboratory. XL designed the retrospective study. YQ was the general manager of the project and designed the research. All authors have read and approved the final version of this manuscript.

## Conflict of Interest

The authors declare that the research was conducted in the absence of any commercial or financial relationships that could be construed as a potential conflict of interest.
