# Supplementary material for: Elevation of Serum Cytokine Profiles and Liver Metabolomic Normalization in Early Convalescence of COVID-19 Patients
Source: Front Med (Lausanne). 2021 Jul 7;8:626633. doi: 10.3389/fmed.2021.626633 (PMC8292617; doi:10.3389/fmed.2021.626633)
Supplement: Supplementary file 1 [file Table_1.DOCX]

**Supporting information**

**Elevation of Serum cytokine profiles and Liver Metabolomic Normalization in Early** **Convalescence of** **COVID-19 Patients**

Yan Lou^1#^, Xiaoying He^1^, Mingxia Deng^1^, Xingjiang Hu^1^, Xi Yang^1^, Lin Liu^1^, Yunzhen Hu^1^, Lingjuan He^1^, Jiali Wang^1^, Li Zhang^1^, Qingwei Zhao^1^, Xiaoyang Lu^1#^, Yunqing Qiu^1#^

1. State Key Laboratory for diagnosis and treatment of infectious diseases, Key Laboratory for Drug Evaluation and Clinical Research of Zhejiang Province, The First Affiliated Hospital, Zhejiang University School of Medicine, Hangzhou 310003, China

^*^Correspondence to: Yunqing Qiu and Yan Lou. State Key Laboratory for diagnosis and treatment of infectious diseases, Key Laboratory for Drug Evaluation and Clinical Research of Zhejiang Province, The First Affiliated Hospital, Zhejiang University School of Medicine, 79 Qingchun Road, Hangzhou 310003, China

Tel: +86 57187236626. Fax: +86 87236626.

E-mail: [qiuyq@zju.edu.cn](mailto:qiuyq@zju.edu.cn) (Yunqing Qiu); [yanlou@zju.edu.cn](mailto:yanlou@zju.edu.cn) (Yan Lou);

**Metabolomic analysis**

Plasma samples were analyzed in both positive and negative electrospray ionization (ESI^+^ and ESI^-^). Plasma samples (100 μL) were precipitated by adding 300 μL of acetonitrile, followed by vigorous shaking for 60 s and centrifugation for 10 min at 13,000 ×g. To monitor the reproducibility and quantitative robustness of the analysis, a pooled sample, serving as the quality control (QC) sample, was prepared by mixing 20 μL of each sample. Then, 100 μL supernatants were transferred to autosampler vials and kept at 10 °C for analysis. The column temperation was set at 40°C. At the beginning of every batch, a ‘blank’ sample containing only the derivatization agents was run to equilibrate the column and assess background ions introduced by sample derivatization. A pooled sample mixed 20 μL of each sample was served as quality control (QC) to monitor the reproducibility and quantitative robustness of the method and instrument. Finally, a 2 μL aliquot of the sample was injected for UPLC/Q-TOF-MS analysis. All analyses were performed using the lockspray, which ensured accuracy and reproducibility.Leucine-enkephalin (5ng/mL) was used as the lock mass generating a reference ion in positive mode at m/z 556.2771 and in negative mode at m/z 554.2615, and introduced by a lockspray at 10μL/min for accurate mass acquisition.

The UPLC Q-TOF system consisted of a Waters Acquity UPLC and Xevo G2-Q-Tof (Waters, Milford, MA, USA), equipped with an ESI source. The chromatographic metabolite separation was achieved on an ACQUITY UPLC HSS T3 column (1.8 μm, 2.1×100 mm) (Waters, Milford, MA, USA). The mobile phase consisted of 0.1% formic acid in water (solvent A) and acetonitrile (solvent B). The MS analysis was operated in both positive (ESI^+^) and negative (ESI^-^) electrospray ionization modes. The gradient elution program was set as follows: 5% B at 0–1 min; 5–60% B at 1–3 min; 60–80% B at 3–7 min; 80–95% B at 7–8.5 min; 95% B at 8.5–13.5 min; and 95%–5% B at 13.5–15 min; 5% B at 15–18 min was used to equilibrate the column. The mass spectrometer parameters for detecting the maximum intensity of precursor ions were optimized as follows: cone gas flow rate: 50 L/h; desolvation gas (N2) flow rate: 800 L/h; capillary voltage: 2.7 kV; sample cone 40 V; source temperature: 100 °C; and desolvation temperature: 400 °C. The MS scan range was 50–1200 mms.

Pre-processing of the obtained raw data, including automatic alignment, peak picking, automatic deconvolution, and global normalization, was achieved by Progenesis QI ver. 2.2 (Nonlinear Dynamic).

For metabolomics, metabolic features with over 80% missingness in a particular group were excluded from the metabolomics dataset. SIMCA-P software (Version 14, Umetrics, Sweden) was employed to analyze the multivariate data matrix. Random forest analysis, a binary classification of viral replication and convalescence phases using differentially regulated metabolites, was performed on R package random forest (version 4.6.14).

**The repeatability and stability of the analytical methods and instruments**

Representative base peak intensity chromatogram was displayed in Supporting Figure S1. Coefficients of variation of the distribution of peaks in the QC samples were displayed in Supporting Figure S2. The peaks of coefficients of variation (CV) below 30 % were more than 84 %, showing good repeatability and stability of the analytical methods and instruments. Additionally, in the principal component analysis score plots (Figure 4A), the QC samples clustered tightly together, which further confirmed the reliability of the present study.

**The validation of OPLS-DA by permutation test**

Permutation testing was performed with an iteration of 200 to check model ability. The result indicated that the OPLS-DA mode was without risk of over-fitting (Figure S3).

**Random forest machine learning**

To investigate the possibility of classifying the virus replication phase and convalescence phase based on the metabolites, a random forest machine learning model was built based on 50 metabolites. An independent set of 13 plasma samples was collected to test the model. All samples including virus replication phase and convalescence phase were correctly identified (Table S9).

**The categorization of liver function abnormality vs liver function normality COVID-19 patients**

Liver injury, mild, moderate, and sever liver injury were defined as occurring of any of the listed abnormal liver function indicators in the corresponding column, respectively (Table S2).

**The categorization of mild vs severe COVID-19 patients by COVID-19 Diagnosis and Treatment Guideline (Trial 5th version)**

According to the Chinese Government Diagnosis and Treatment Guideline (Trial 5th version) (Medicine, 2020), COVID-19 patients are classified into four subgroups: 1) Mild: mild symptoms without pneumonia; 2) Typical: fever or respiratory tract symptoms with pneumonia; 3) Severe: fulfill any of the three criteria: respiratory distress, respiratory rate ≥ 30 times/min; means oxygen saturation ≤ 93% in resting state; arterial blood oxygen partial pressure/oxygen concentration ≤ 300 mmHg (1 mmHg = 0.133 kPa); 4) Critical: fulfill any of the three criteria: respiratory failure and require mechanical ventilation; shock incidence; admission to ICU with other organ failure. In this study, we included both severe and non-severe patients, with the latter composed of mild and typical COVID-19 patients.

**IL-6, IL-10, IL-4 signal pathways**

IL-6 can signal through three main pathways referred to JAK-STAT3，JAK–SHP-2–MAPK and PI3K/AKT pathways (1). IL-6 plays a critically pro-inflammatory role by activation of these pathways (2). The elevated level of 4-hydroxycinnamic acid (3), Ganglioside GM3 (4), L-Phenylalanine (5), Linoleic acid (6), L-Carnitine (7, 8), Oleic acid (9, 10), Stearic acid (11, 12), DHEAS (13), DHA (14-16), PA (17, 18), PC (19, 20), PI (21, 22), LysoPC (23) and L-Acetylcarnitine (24) inhibited JAK-STAT3，JAK–SHP-2–MAPK and PI3K/AKT pathways (3), while the decreased level of palmitic acid (25-27), Lactosylceramide (28, 29) and phytosphingosine (30) also inhibited aforementioned pathways in the convalescence. Thus, the changes in the levels of these metabolites are the manifestations of hyper-inhibition of JAK-STAT3, JAK–SHP-2–MAPK and PI3K/AKT pathways during the convalescence.

IL-10 is a major anti-inflammatory cytokine secreted by macrophages and exerts it effect via the JAK-STAT3 and MAPK pathways (31). Palmitic acid (26) and DHA (14) levels were down-regulated and up-regulated, respectively, both of which could inhibit STAT3 signaling to promote liver repair. The elevated level of sphingosine 1-phosphate (32), 4-hydroxycinnamic acid (3), ganglioside GM3 (4), L-tryptophan (33), L-carnitine (34), oleic acid (35), kinetin (36), DHEAS (13), DHA (37), DPA (38), PC(20) and PI (21) inhibited MAPK pathway, while the decreased level of palmitic acid (39), lactosylceramide (40) and phytosphingosine (41) also inhibited aforementioned pathways in the convalescence. Thus, the changes in the levels of these metabolites are the manifestations of hyper-inhibition of JAK-STAT3 and MAPK pathways during the convalescence.

IL-4 is one of the best-known anti-inflammatory cytokines due to its widespread biological roles mediated mainly via JAK-STAT6 pathway (42). Both up-regulated metabolites (Sphingosine 1-phosphate (43), LPC (44), DHEAS (45) and DHA (46)) and down regulated metabolites (palmitic acid (47) and Lactosylceramide (48)) ,suggested that the production of IL-4 by T cells was activated in the convalescence.

**Reference**

1. Kang S, Tanaka T, Narazaki M, Kishimoto T. Targeting Interleukin-6 Signaling in Clinic. Immunity 2019;50:1007-1023.

2. Schett G. Physiological effects of modulating the interleukin-6 axis. Rheumatology (Oxford) 2018;57:ii43-ii50.

3. Park SH, Ko JW, Shin NR, Shin DH, Cho YK, Seo CS, Kim JC, et al. 4-Hydroxycinnamic acid protects mice from cigarette smoke-induced pulmonary inflammation via MAPK pathways. Food Chem Toxicol 2017;110:151-155.

4. Park J, Kwak CH, Ha SH, Kwon KM, Abekura F, Cho SH, Chang YC, et al. Ganglioside GM3 suppresses lipopolysaccharide-induced inflammatory responses in rAW 264.7 macrophage cells through NF-kappaB, AP-1, and MAPKs signaling. J Cell Biochem 2018;119:1173-1182.

5. Li D, Gu X, Lu L, Liang L. Effects of phenylalanine on the survival and neurite outgrowth of rat cortical neurons in primary cultures: possible involvement of brain-derived neurotrophic factor. Mol Cell Biochem 2010;339:1-7.

6. Marei WF, Wathes DC, Fouladi-Nashta AA. Impact of linoleic acid on bovine oocyte maturation and embryo development. Reproduction 2010;139:979-988.

7. Ge P, Cui Y, Liu F, Luan J, Zhou X, Han J. L-carnitine affects osteoblast differentiation in NIH3T3 fibroblasts by the IGF-1/PI3K/Akt signalling pathway. Biosci Trends 2015;9:42-48.

8. Baek JE, Yang WS, Chang JW, Kim SB, Park SK, Park JS, Lee SK. Fatty acid-bearing albumin induces VCAM-1 expression through c-Src kinase-AP-1/NF-kB pathways: effect of L-carnitine. Kidney Blood Press Res 2010;33:72-84.

9. Jiang L, Wang W, He Q, Wu Y, Lu Z, Sun J, Liu Z, et al. Oleic acid induces apoptosis and autophagy in the treatment of Tongue Squamous cell carcinomas. Sci Rep 2017;7:11277.

10. Liu Y, Yu Q, Chen Y. Effect of silibinin on CFLAR-JNK pathway in oleic acid-treated HepG2 cells. Biomed Pharmacother 2018;108:716-723.

11. Sramek J, Nemcova-Furstova V, Pavlikova N, Kovar J. Effect of Saturated Stearic Acid on MAP Kinase and ER Stress Signaling Pathways during Apoptosis Induction in Human Pancreatic beta-Cells Is Inhibited by Unsaturated Oleic Acid. Int J Mol Sci 2017;18.

12. Meng Y, Yuan C, Zhang J, Zhang F, Fu Q, Zhu X, Shu G, et al. Stearic acid suppresses mammary gland development by inhibiting PI3K/Akt signaling pathway through GPR120 in pubertal mice. Biochem Biophys Res Commun 2017;491:192-197.

13. Ziegler CG, Langbein H, Krug AW, Ludwig B, Eisenhofer G, Ehrhart-Bornstein M, Bornstein SR. Direct effect of dehydroepiandrosterone sulfate (DHEAS) on PC-12 cell differentiation processes. Mol Cell Endocrinol 2011;336:149-155.

14. Tasaki S, Horiguchi A, Asano T, Ito K, Asano T, Asakura H. Docosahexaenoic acid inhibits the phosphorylation of STAT3 and the growth and invasion of renal cancer cells. Exp Ther Med 2017;14:1146-1152.

15. Yin Y, Sui C, Meng F, Ma P, Jiang Y. The omega-3 polyunsaturated fatty acid docosahexaenoic acid inhibits proliferation and progression of non-small cell lung cancer cells through the reactive oxygen species-mediated inactivation of the PI3K /Akt pathway. Lipids Health Dis 2017;16:87.

16. Kim HJ, Ohk B, Yoon HJ, Kang WY, Seong SJ, Kim SY, Yoon YR. Docosahexaenoic acid signaling attenuates the proliferation and differentiation of bone marrow-derived osteoclast precursors and promotes apoptosis in mature osteoclasts. Cell Signal 2017;29:226-232.

17. Lee SY, Lee YY, Choi JS, Yoon MS, Han JS. Phosphatidic acid induces decidualization by stimulating Akt-PP2A binding in human endometrial stromal cells. FEBS J 2016;283:4163-4175.

18. Ouro A, Arana L, Rivera IG, Ordonez M, Gomez-Larrauri A, Presa N, Simon J, et al. Phosphatidic acid inhibits ceramide 1-phosphate-stimulated macrophage migration. Biochem Pharmacol 2014;92:642-650.

19. Koeberle A, Shindou H, Koeberle SC, Laufer SA, Shimizu T, Werz O. Arachidonoyl-phosphatidylcholine oscillates during the cell cycle and counteracts proliferation by suppressing Akt membrane binding. Proc Natl Acad Sci U S A 2013;110:2546-2551.

20. Treede I, Braun A, Sparla R, Kuhnel M, Giese T, Turner JR, Anes E, et al. Anti-inflammatory effects of phosphatidylcholine. J Biol Chem 2007;282:27155-27164.

21. van Dieren JM, Simons-Oosterhuis Y, Raatgeep HC, Lindenbergh-Kortleve DJ, Lambers ME, van der Woude CJ, Kuipers EJ, et al. Anti-inflammatory actions of phosphatidylinositol. Eur J Immunol 2011;41:1047-1057.

22. Matsunaga N, Shimazawa M, Otsubo K, Hara H. Phosphatidylinositol inhibits vascular endothelial growth factor-A--induced migration of human umbilical vein endothelial cells. J Pharmacol Sci 2008;106:128-135.

23. Rikitake Y, Kawashima S, Yamashita T, Ueyama T, Ishido S, Hotta H, Hirata K, et al. Lysophosphatidylcholine inhibits endothelial cell migration and proliferation via inhibition of the extracellular signal-regulated kinase pathway. Arterioscler Thromb Vasc Biol 2000;20:1006-1012.

24. Aguer C, McCoin CS, Knotts TA, Thrush AB, Ono-Moore K, McPherson R, Dent R, et al. Acylcarnitines: potential implications for skeletal muscle insulin resistance. FASEB J 2015;29:336-345.

25. Zhou T, Wang G, Lyu Y, Wang L, Zuo S, Zou J, Sun L, et al. Upregulation of SLAMF3 on human T cells is induced by palmitic acid through the STAT5-PI3K/Akt pathway and features the chronic inflammatory profiles of type 2 diabetes. Cell Death Dis 2019;10:559.

26. Zhou BR, Zhang JA, Zhang Q, Permatasari F, Xu Y, Wu D, Yin ZQ, et al. Palmitic acid induces production of proinflammatory cytokines interleukin-6, interleukin-1beta, and tumor necrosis factor-alpha via a NF-kappaB-dependent mechanism in HaCaT keratinocytes. Mediators Inflamm 2013;2013:530429.

27. Liang Z, Yuan Z, Guo J, Wu J, Yi J, Deng J, Shan Y. Ganoderma lucidum Polysaccharides Prevent Palmitic Acid-Evoked Apoptosis and Autophagy in Intestinal Porcine Epithelial Cell Line via Restoration of Mitochondrial Function and Regulation of MAPK and AMPK/Akt/mTOR Signaling Pathway. Int J Mol Sci 2019;20.

28. Bhunia AK, Han H, Snowden A, Chatterjee S. Lactosylceramide stimulates Ras-GTP loading, kinases (MEK, Raf), p44 mitogen-activated protein kinase, and c-fos expression in human aortic smooth muscle cells. J Biol Chem 1996;271:10660-10666.

29. Chatterjee S, Pandey A. The Yin and Yang of lactosylceramide metabolism: implications in cell function. Biochim Biophys Acta 2008;1780:370-382.

30. Park KM, Wang JW, Yoo YM, Choi MJ, Hwang KC, Jeung EB, Jeong YW, et al. Sphingosine-1-phosphate (S1P) analog phytosphingosine-1-phosphate (P1P) improves the in vitro maturation efficiency of porcine oocytes via regulation of oxidative stress and apoptosis. Mol Reprod Dev 2019;86:1705-1719.

31. Brockmann L, Gagliani N, Steglich B, Giannou AD, Kempski J, Pelczar P, Geffken M, et al. IL-10 Receptor Signaling Is Essential for TR1 Cell Function In Vivo. J Immunol 2017;198:1130-1141.

32. Stradner MH, Gruber G, Angerer H, Huber V, Setznagl D, Kremser ML, Moazedi-Furst FC, et al. Sphingosine 1-phosphate counteracts the effects of interleukin-1beta in human chondrocytes. Arthritis Rheum 2013;65:2113-2122.

33. Mine Y, Zhang H. Calcium-sensing receptor (CaSR)-mediated anti-inflammatory effects of L-amino acids in intestinal epithelial cells. J Agric Food Chem 2015;63:9987-9995.

34. Salama SA, Arab HH, Omar HA, Gad HS, Abd-Allah GM, Maghrabi IA, Al Robaian MM. L-carnitine mitigates UVA-induced skin tissue injury in rats through downregulation of oxidative stress, p38/c-Fos signaling, and the proinflammatory cytokines. Chem Biol Interact 2018;285:40-47.

35. Reyes-Quiroz ME, Alba G, Saenz J, Santa-Maria C, Geniz I, Jimenez J, Ramirez R, et al. Oleic acid modulates mRNA expression of liver X receptor (LXR) and its target genes ABCA1 and SREBP1c in human neutrophils. Eur J Nutr 2014;53:1707-1717.

36. Wei Y, Liu D, Zheng Y, Hao C, Li H, Ouyang W. Neuroprotective Effects of Kinetin Against Glutamate-Induced Oxidative Cytotoxicity in HT22 Cells: Involvement of Nrf2 and Heme Oxygenase-1. Neurotox Res 2018;33:725-737.

37. Yamagata K, Suzuki S, Tagami M. Docosahexaenoic acid prevented tumor necrosis factor alpha-induced endothelial dysfunction and senescence. Prostaglandins Leukot Essent Fatty Acids 2016;104:11-18.

38. Morin C, Hiram R, Rousseau E, Blier PU, Fortin S. Docosapentaenoic acid monoacylglyceride reduces inflammation and vascular remodeling in experimental pulmonary hypertension. Am J Physiol Heart Circ Physiol 2014;307:H574-586.

39. Kobayashi M, Yoshino O, Nakashima A, Ito M, Nishio K, Ono Y, Kusabiraki T, et al. Inhibition of autophagy in theca cells induces CYP17A1 and PAI-1 expression via ROS/p38 and JNK signalling during the development of polycystic ovary syndrome. Mol Cell Endocrinol 2020;508:110792.

40. Mishra S, Chatterjee S. Lactosylceramide promotes hypertrophy through ROS generation and activation of ERK1/2 in cardiomyocytes. Glycobiology 2014;24:518-531.

41. Nagahara Y, Kawakami K, Sikandan A, Yagi D, Nishikawa R, Shinomiya T. Sphingoid Base-Upregulated Caspase-14 Expression Involves MAPK. Biol Pharm Bull 2018;41:743-748.

42. Busch-Dienstfertig M, Gonzalez-Rodriguez S. IL-4, JAK-STAT signaling, and pain. JAKSTAT 2013;2:e27638.

43. Wang W, Huang MC, Goetzl EJ. Type 1 sphingosine 1-phosphate G protein-coupled receptor (S1P1) mediation of enhanced IL-4 generation by CD4 T cells from S1P1 transgenic mice. J Immunol 2007;178:4885-4890.

44. Hung ND, Sok DE, Kim MR. Prevention of 1-palmitoyl lysophosphatidylcholine-induced inflammation by polyunsaturated acyl lysophosphatidylcholine. Inflamm Res 2012;61:473-483.

45. Lee J, Sepulveda RT, Jiang S, Zhang Z, Inserra P, Zhang Y, Hosseini S, et al. Immune dysfunction during alcohol consumption and murine AIDS: the protective role of dehydroepiandrosterone sulfate. Alcohol Clin Exp Res 1999;23:856-862.

46. Attakpa E, Hichami A, Simonin AM, Sanson EG, Dramane KL, Khan NA. Docosahexaenoic acid modulates the expression of T-bet and GATA-3 transcription factors, independently of PPARalpha, through suppression of MAP kinase activation. Biochimie 2009;91:1359-1365.

47. Ko JS, Koh JM, So JS, Jeon YK, Kim HY, Chung DH. Palmitate inhibits arthritis by inducing t-bet and gata-3 mRNA degradation in iNKT cells via IRE1alpha-dependent decay. Sci Rep 2017;7:14940.

48. Lai AC, Chi PY, Thio CL, Han YC, Kao HN, Hsieh HW, Gervay-Hague J, et al. alpha-Lactosylceramide Protects Against iNKT-Mediated Murine Airway Hyperreactivity and Liver Injury Through Competitive Inhibition of Cd1d Binding. Front Chem 2019;7:811.

**Figure legends**

**Figure S1** Representative base peak intensity chromatogram.

**Figure S2** CV distribution of peaks in metabolomic dataset. The number of peaks and accumulative percentage of peaks in corresponding CV interval were expressed as black and red column, respectively.

**Figure S3** Internal validation of the OPLS-DA model by 200 permutation test.

**Figure S4** The correlation between the selected metabolites and cytokines was expressed by heatmap with significance indicated by the asterisks (p value: ***, <0.001; **, <0.01; *, <0.05), the colors changing from red (positive correlation) to blue (negative correlation)

**Figure S5** Change of expression level metabolites related to the liver function indicators

**Figure S6** Metabolomic profiling of COVID-19 and Healthy volunteers plasma. PCA and OPLS-DA plot of COVID-19 patients in convalescence phase and healthy volunteers, the colors changing from red (up-regulated metabolites) to blue (down-regulated metabolites).

**Figure S7** Heatmap of differential metablites in convalescent patients and healthy volunteers, the colors changing from red to blue.

Figure S1


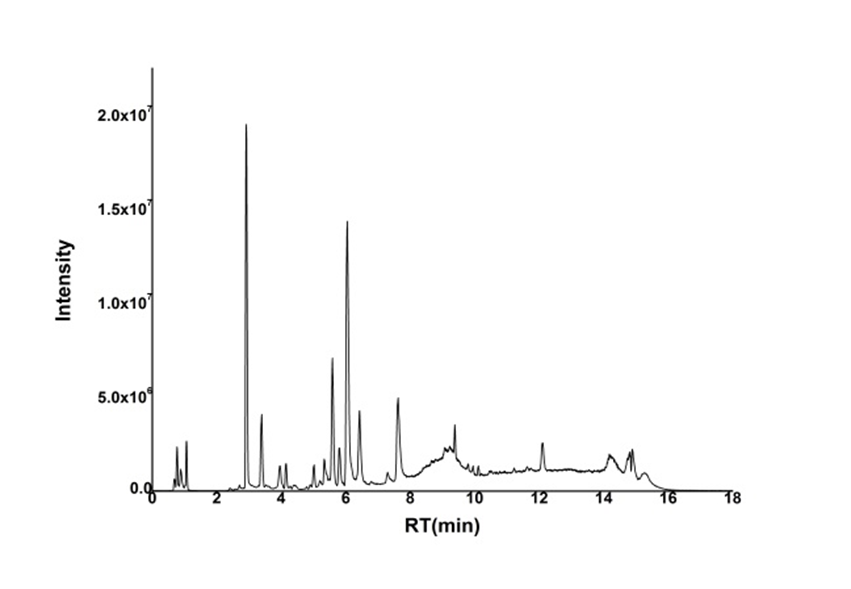


Figure S2


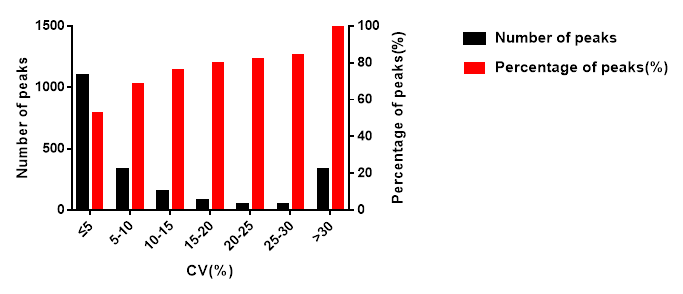


Figure S3


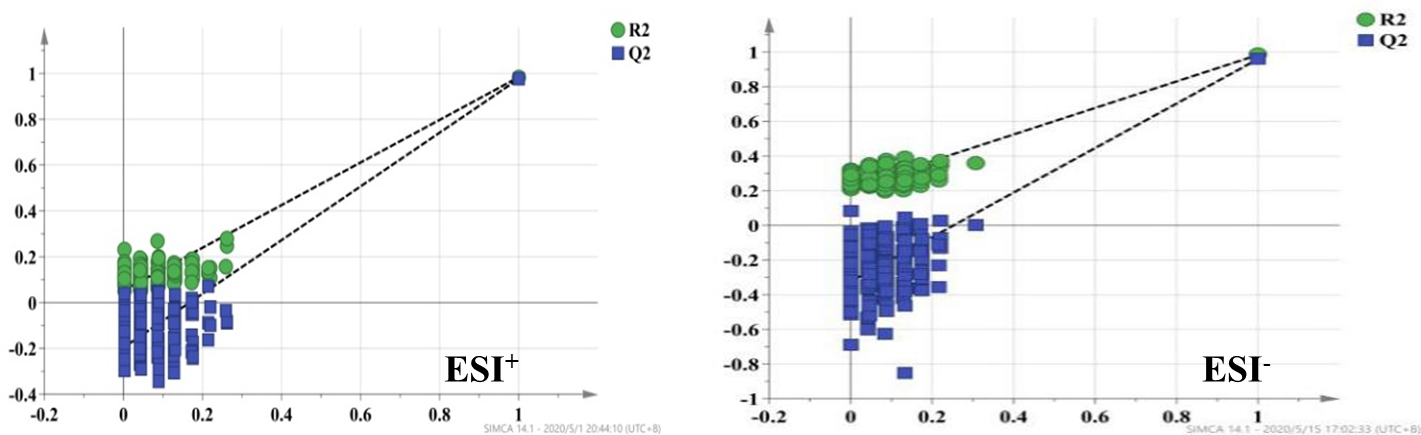


Figure S4


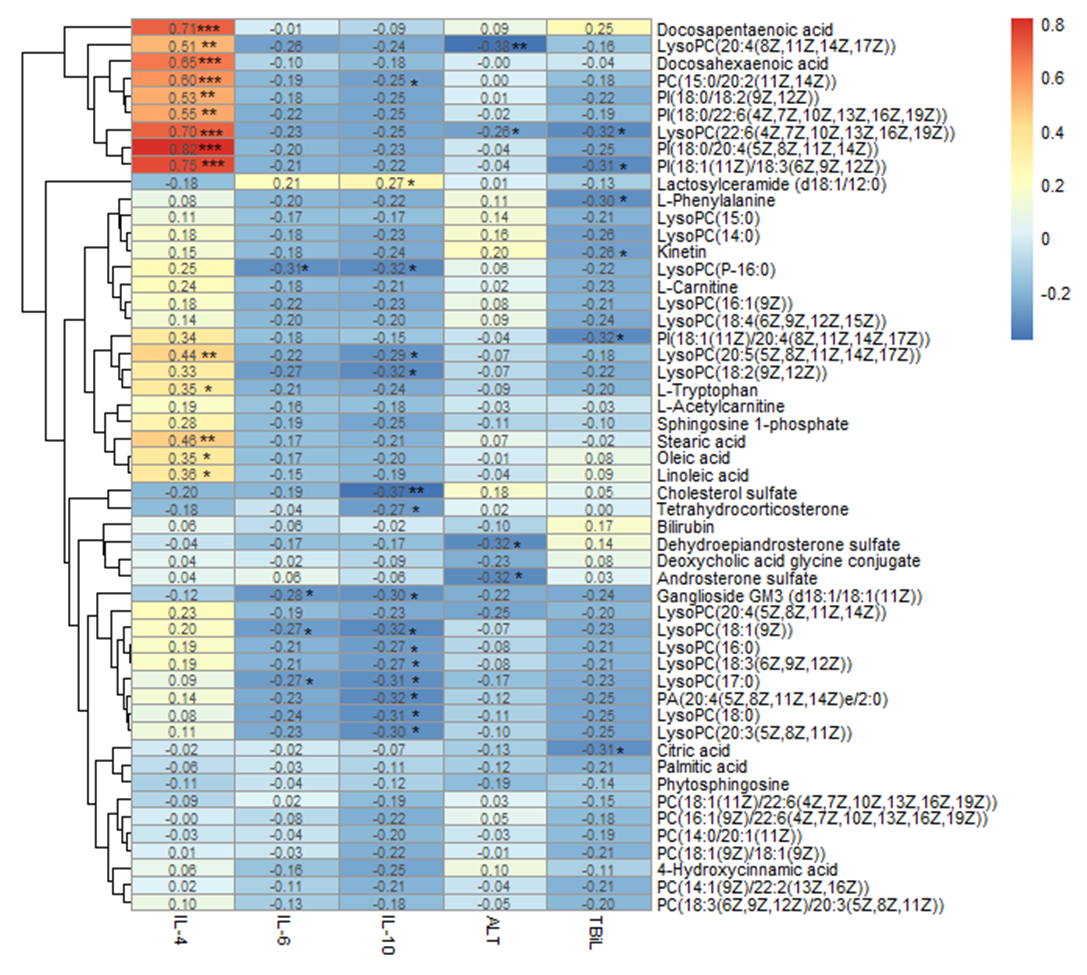


Figure S5


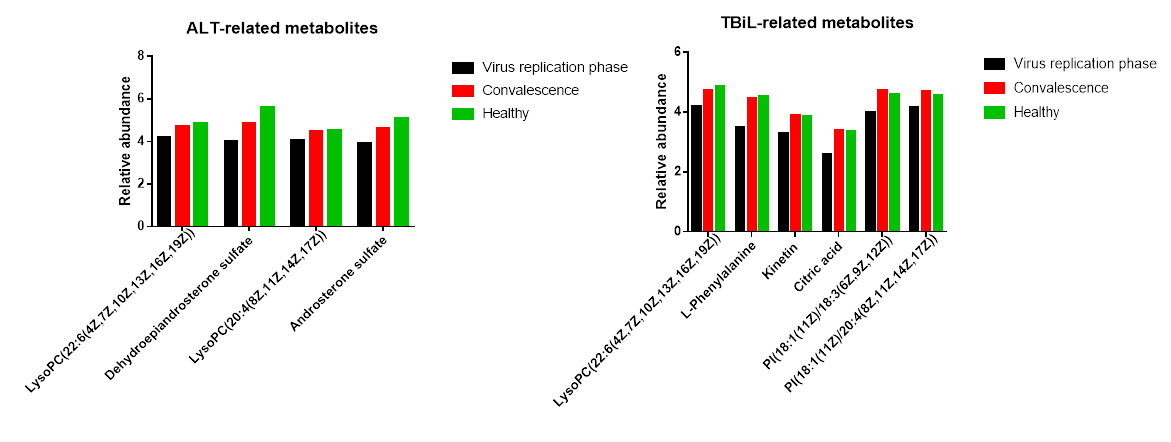


Figure S6


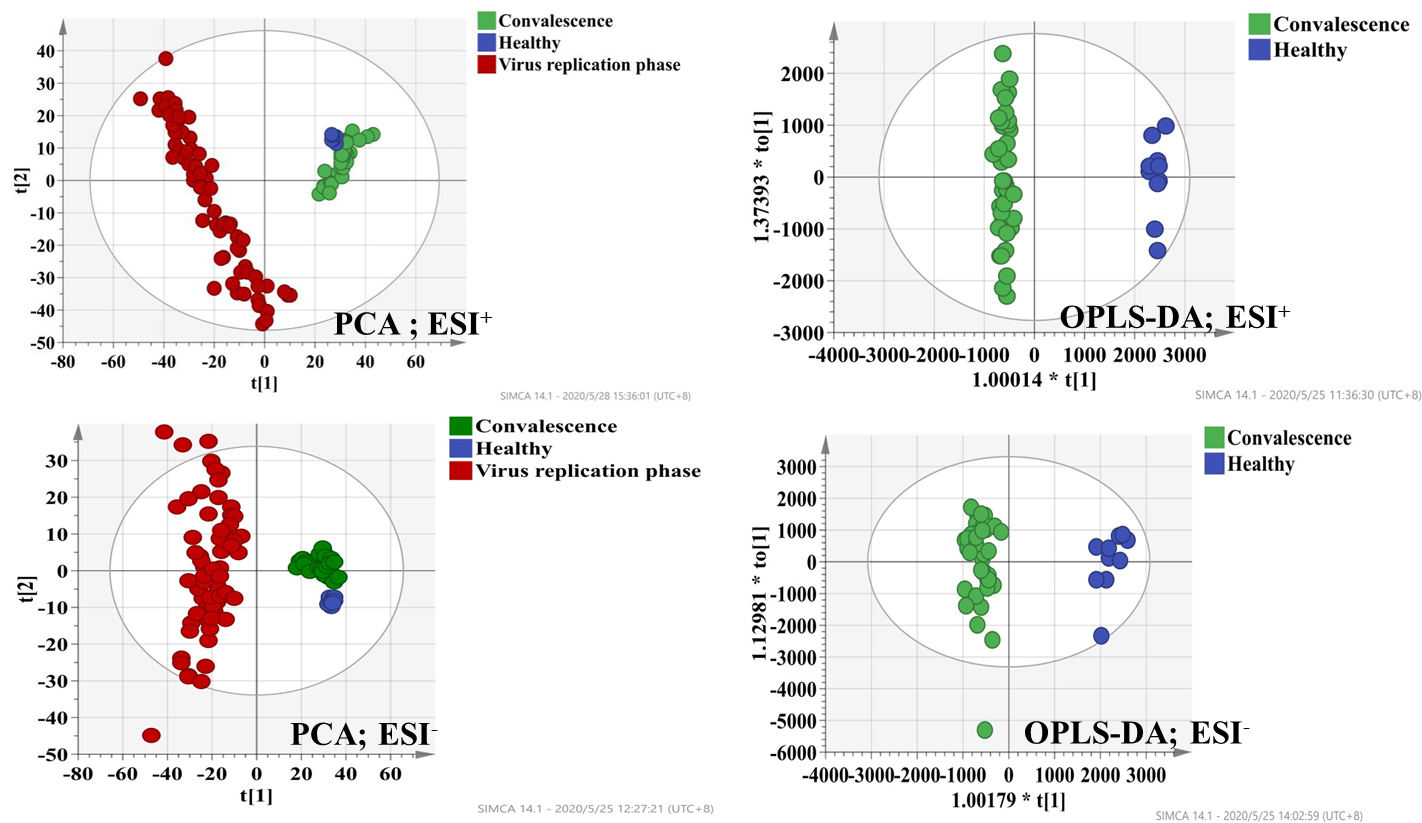


Figure S7


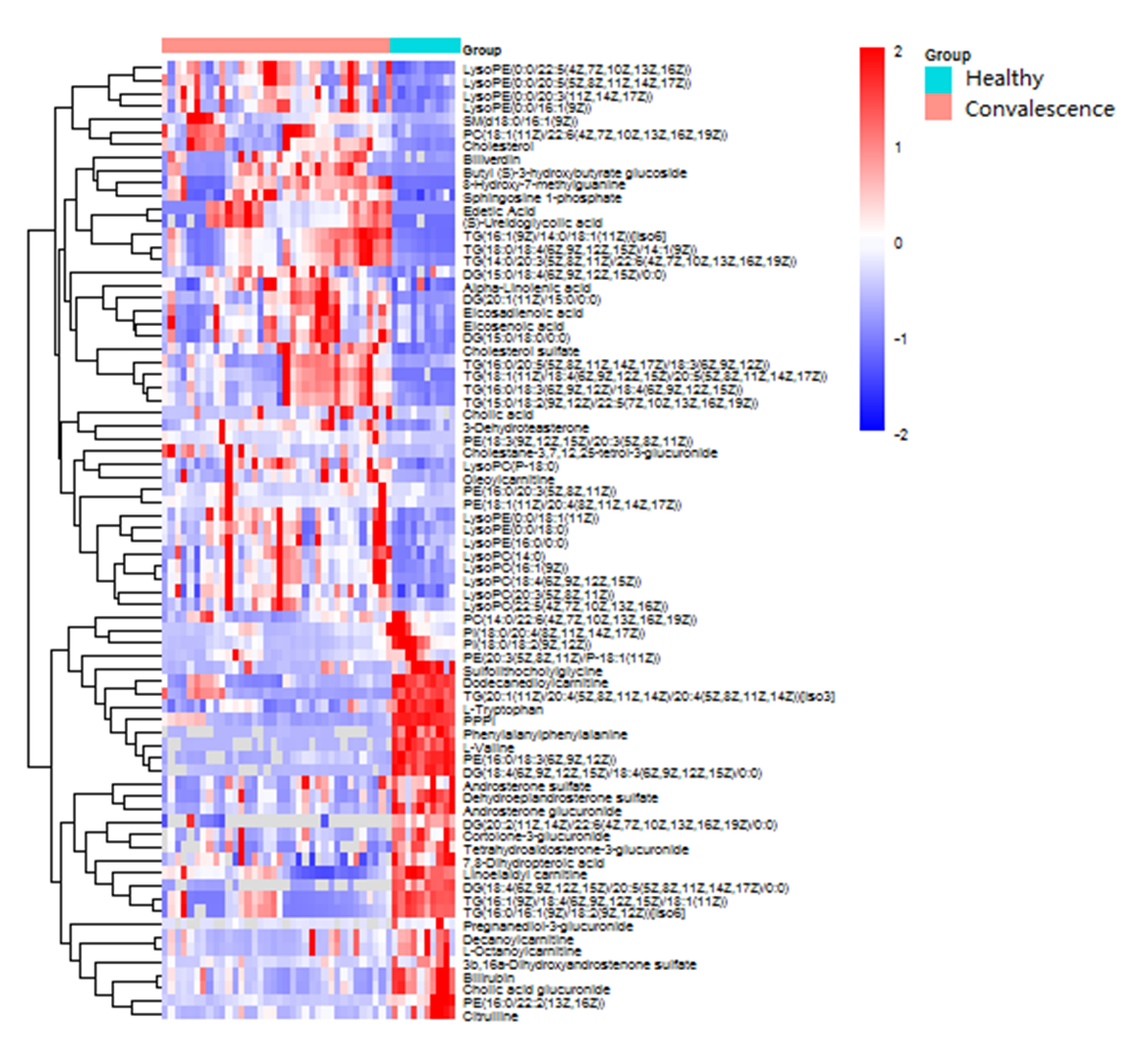


TableS1 The normal reference range for clinical biochemical indicators and cytokines

| Biochemical indicators | ALT | AST | ALP | TBiL* |  |  |
| --- | --- | --- | --- | --- | --- | --- |
| Reference(U/L) | 5-40 | 8-40 | 40-150 | 0.0-21.0(μmol/L) |  |  |
| Cytokines | IL-2 | IL-4 | IL-6 | IL-10 | TNF-α | IFN-γ |
| Reference(pg/mL) | <4.13 | <8.37 | <6.61 | <2.31 | <33.27 | <20.06 |

*The unit of TBiL is μmol/L

Table S2 Definition of liver injury

| Indicators | Liver injury | Mild liver injury | Moderate liver injury | Sever liver injury |
| --- | --- | --- | --- | --- |
| ALT | >1 ULN | >1-3 ULN | >3-5 ULN | >5 ULN |
| AST | >1 ULN | >1-3 ULN | >3-5 ULN | >5 ULN |
| ALP | >1 ULN | >1-2.5 ULN | >2.5-5 ULN | >5 ULN |
| TBiL | >1 ULN | >1-1.5 ULN | >1.5-3 ULN | >3 ULN |

Note: COVID-19-associated liver injury is defined as any liver damage occurring during COVID-19 disease progression and treatment in patients

Table S3 The abnormal range of indicators

| Indicators | Abnormal (min-max) |
| --- | --- |
| ALT | 112(47-1122) |
| AST | 58.5(41-229) |
| ALP | 148(133-179) |
| TBiL | 28.73(17.4-57.7) |

Table S4 The dynamic changes of IL-6, IL-10, IL-4, TNF-α, IFN-γ and IL-2 in virus replication phase and convalescence.

|  | Virus replication phase | | | | | Convalescence | | Virus replication phase | | | | | Convalescence | | |
| --- | --- | --- | --- | --- | --- | --- | --- | --- | --- | --- | --- | --- | --- | --- | --- |
| Time points | 4-6 | 7-9 | 10-12 | 13-15 | ≥16 | 7-9 | 13-15 | 4-6 | 7-9 | 10-12 | 13-15 | ≥16 | 7-9 | 13-15 | |
|  | IL-6 (N=81) | | | | | | | IL-10 (N=81) | | | | | | | |
| Mean  (SD) | 50.16  (74.82) | 78.70  (147.06) | 85.23  (200.66) | 33.17  (67.79) | 26.70  (68.85) | 8.17  (7.98) | 10.59  (7.92) | 6.18  (5.89) | 6.75  (5.75) | 6.03  (4.50) | 4.10  (3.10) | 3.18  (4.33) | 1.65  (1.03) | 1.88  (1.50) | |
| Median | 24.84 | 23.72 | 24.16 | 12.18 | 10.64 | 4.89 | 7.95 | 3.66 | 5.84 | 5.03 | 3.2 | 2.26 | 1.25 | 1.40 | |
| min | 4.00 | 1.32 | 1.78 | 0.46 | 1.42 | 1.43 | 2.81 | 0.93 | 0.79 | 0.80 | 0.79 | 0.67 | 0.69 | 0.67 | |
| max | 286.70 | 828.11 | 1436.82 | 501.83 | 744.36 | 40.25 | 37.77 | 20.70 | 26.11 | 20.61 | 18.89 | 56.87 | 5.41 | 8.42 | |
|  | IL-4 (N=26) | | | | | | | TNF-α (N=68) | | | | | | | |
| Mean  (SD) | 1.28  (0.38) | 1.45  (1.06) | 1.17  (0.98) | 2.04  (0.87) | 2.62  (75.31) | 5.97  (6.15) | 6.98  (6.32) | 36.28  (39.39) | 29.30  (38.00) | 30.16  (20.16) | 38.39  (34.50) | 27.73  (19.48) | 40.80  (39.80) | 50.44  (47.39) | |
| Median | 1.22 | 1.01 | 0.76 | 2.09 | 1.80 | 2.19 | 6.48 | 17.69 | 17.83 | 19.93 | 24.24 | 23.29 | 23.27 | 37.96 | |
| min | 0.93 | 0.29 | 0.06 | 0.20 | 1.05 | 1.57 | 1.15 | 1.18 | 0.10 | 0.04 | 0.26 | 6.69 | 6.84 | 6.82 | |
| max | 1.77 | 3.47 | 3.08 | 3.45 | 22.1 | 17.97 | 23.24 | 117.69 | 196.06 | 115.20 | 156.74 | 113.23 | 157.06 | 212.65 | |
|  | IFN-γ (N=74) | | | | | | | IL-2 (N=44) | | | | | | | |
| Mean  (SD) | 19.73  (20.24) | 22.65  (29.51) | 24.86  (46.16) | 13.68  (13.22) | 28.84  (64.39) | 20.43  (24.32) | 23.94  (27.77) | 1.51  (0.67) | 2.09  (2.27) | 1.95  (2.28) | 2.18  (1.19) | 1.90  (1.10) | 2.12  (1.27) | | 2.79  (2.67) |
| Median | 9.20 | 9.96 | 11.22 | 10.00 | 12.31 | 10.98 | 14.33 | 1.36 | 1.32 | 1.32 | 1.95 | 1.56 | 1.69 | | 1.80 |
| min | 2.51 | 0.62 | 1.47 | 2.12 | 2.84 | 2.90 | 3.39 | 0.65 | 0.20 | 0.83 | 0.76 | 0.71 | 1.04 | | 0.23 |
| max | 63.91 | 127.06 | 310.12 | 64.37 | 477.6 | 125.63 | 123.76 | 2.57 | 9.88 | 13.04 | 5.05 | 5.49 | 5.35 | | 9.54 |

Table S5 Cytokine (IL-6, IL-10) comparison of patients with different ages, different liver function and different severity of infection in virus replication phase

| Time pionts (days) | | 4-6 | 7-9 | 10-12 | 13-15 | ≥16 |
| --- | --- | --- | --- | --- | --- | --- |
| IL-6 | **Different Ages** | | | | | |
|  | ＜70 | 21.93(5.67-50.46) | 14.27(1.32-342.21) | 19.46(1.78-495.97) | 9.35(0.46-501.83) | 7.72(1.42-475.46) |
|  | ≥70 | 47.18(4.00-286.70) | 70.74(3.14-828.11) | 44.74(2.03-1436.82) | 16.05(4.27-372.82) | 24.46(1.59-744.36) |
|  | P | 0.162 | 0.070 | 0.010 | 0.001 | ＜0.001 |
|  | **Liver function** | | | | | |
|  | Liver impaired | 32.69(4.00-286.70) | 43.01(3.34-828.11) | 34.26(2.03-1436.82) | 14.81(2.32-501.83) | 12.65(1.95-744.36) |
|  | Liver normal | 14.79(5.67-34.73) | 11.87(1.32-142.52) | 14.08(1.78-495.97) | 7.49(0.46-88.65) | 6.71(1.42-110.04) |
|  | P | 0.123 | 0.034 | 0.037 | 0.001 | 0.001 |
|  | **Severity** | | | | | |
|  | Mild patients | 21.05(5.67- 50.46) | 14.13(1.32- 142.52) | 15.72(4.13-90.00) | 5.78(2.97-33.98) | 6.73(1.42-50.08) |
|  | Severe patients | 28.59(4.00-286.70) | 33.95(3.14-828.11) | 24.24(1.78-1436.82) | 11.97(0.46- 372.82) | 11.08(1.56- 744.36) |
|  | P | 0.499 | 0.202 | 0.482 | 0.034 | 0.034 |
| IL-10 | **Different Ages** | | | | | |
|  | ＜70 | 2.53(0.93-9.97) | 3.94(0.79-21.94) | 4.30(0.87-20.61) | 2.99(0.79-17.12) | 1.95(0.67-56.87) |
|  | ≥70 | 6.03(3.32-14.22) | 8.64(0.85-26.11) | 5.52(0.80-19.35) | 4.29(1.43-18.89) | 3.59(1.22-13.33) |
|  | P | 0.159 | 0.023 | 0.151 | 0.001 | ＜0.001 |
|  | **Liver function** | | | | | |
|  | Liver impaired | 4.35(1.19-20.70) | 6.25(0.79-26.11) | 5.48(1.05-19.35) | 3.46(0.91-18.89) | 2.35(0.67-56.87) |
|  | Liver normal | 2.16(0.93-7.91) | 2.90(0.85-8.06) | 3.37(0.80-20.61) | 3.04(0.79-11.10) | 2.00(0.81-7.53) |
|  | P | 0.165 | 0.004 | 0.043 | 0.093 | 0.015 |
|  | **Severity** | | | | | |
|  | Mild patients | 1.33(0.93-3.66) | 3.40(0.92-12.24) | 3.29(1.14-13.90) | 2.31(0.79-4.02) | 1.50(0.81-4.11) |
|  | Severe patients | 4.95(1.19-20.70) | 6.47(0.79-25.49) | 4.89(0.80-71.25) | 3.40(0.91-51.99) | 2.42(0.67-56.87) |
|  | P | 0.083 | 0.055 | 0.430 | 0.014 | ＜0.001 |
| Data was expressed as median (min-max); P value was calculated using one-way ANOVA or Mann-Whitney U test | | | | | | |

Table S6 The correlation between cytokine (IL-4, IL-6, IL-10) and liver function indicators

|  | ALT | | TBiL | | AST | | ALP | |
| --- | --- | --- | --- | --- | --- | --- | --- | --- |
|  | r | P value | r | P value | r | P value | r | P value |
| IL-4(N=432) | -0.161** | 0.001 | 0.020 | 0.686 | 0.006 | 0.896 | -0.053 | 0.268 |
| IL-6(N=427) | -0.096* | 0.048 | 0.164** | 0.001 | 0.020 | 0.686 | -0.010 | 0.829 |
| IL-10(N=410) | -0.072 | 0.147 | 0.175*** | 0.000 | 0.042 | 0.393 | -0.164** | 0.001 |
| TNF-α (N=320) | -0.025 | 0.655 | -0.084 | 0.134 | -0.017 | 0.769 | -0.101 | 0.071 |
| IFN-γ(N=335) | -0.028 | 0.606 | -0.094 | 0.086 | -0.024 | 0.658 | 0.065 | 0.236 |

P value was calculated by Spearman correlation with significance indicated by the asterisks (p value: ***, <0.001; **, <0.01; *, <0.05)Table S6 The information of all the metabolite biomarkers

Table S7 The information of all the metabolite biomarkers

| Description | m/z | ID | P | FC | VIP | AUC |
| --- | --- | --- | --- | --- | --- | --- |
| LysoPC(16:0) | 496.3408 | HMDB10382 | <0.001 | 0.2368 | 25.9971 | 0.997 |
| LysoPC(18:2(9Z,12Z)) | 520.3404 | HMDB10386 | <0.001 | 0.2259 | 14.4153 | 0.987 |
| PC(14:0/20:1(11Z)) | 760.5825 | HMDB07879 | <0.001 | 0.2922 | 13.4676 | 0.984 |
| LysoPC(18:0) | 524.3717 | HMDB10384 | <0.001 | 0.3208 | 13.3763 | 0.985 |
| PC(18:1(9Z)/18:1(9Z)) | 786.5995 | HMDB00593 | <0.001 | 0.2945 | 12.5590 | 0.988 |
| LysoPC(18:1(9Z)) | 522.3559 | HMDB02815 | <0.001 | 0.2716 | 12.4223 | 0.980 |
| LysoPC(18:3(6Z,9Z,12Z)) | 518.3218 | HMDB10387 | <0.001 | 0.2293 | 10.2793 | 1.000 |
| PC(14:1(9Z)/22:2(13Z,16Z)) | 784.5827 | HMDB07921 | <0.001 | 0.3383 | 10.1862 | 0.973 |
| LysoPC(20:3(5Z,8Z,11Z)) | 546.3530 | HMDB10393 | <0.001 | 0.2840 | 6.6839 | 0.997 |
| LysoPC(20:4(5Z,8Z,11Z,14Z)) | 544.3392 | HMDB10395 | <0.001 | 0.2695 | 6.5853 | 0.987 |
| PC(16:1(9Z)/22:6(4Z,7Z,10Z,13Z,16Z,19Z)) | 804.5518 | HMDB08023 | <0.001 | 0.3838 | 5.3296 | 0.931 |
| LysoPC(16:1(9Z)) | 494.3241 | HMDB10383 | <0.001 | 0.2509 | 3.9685 | 0.980 |
| PC(18:1(11Z)/22:6(4Z,7Z,10Z,13Z,16Z,19Z)) | 832.5815 | HMDB08090 | <0.001 | 0.4324 | 3.2368 | 0.907 |
| PC(18:3(6Z,9Z,12Z)/20:3(5Z,8Z,11Z)) | 806.5677 | HMDB08178 | <0.001 | 0.4041 | 3.0557 | 0.960 |
| LysoPC(14:0) | 468.3084 | HMDB10379 | <0.001 | 0.1892 | 2.8469 | 0.973 |
| LysoPC(20:5(5Z,8Z,11Z,14Z,17Z)) | 542.3229 | HMDB10397 | <0.001 | 0.2629 | 2.5858 | 0.961 |
| LysoPC(22:6(4Z,7Z,10Z,13Z,16Z,19Z)) | 568.3394 | HMDB10404 | <0.001 | 0.3836 | 2.0555 | 0.952 |
| LysoPC(15:0) | 482.3242 | HMDB10381 | <0.001 | 0.3250 | 1.4731 | 0.931 |
| LysoPC(P-16:0) | 480.3445 | HMDB10407 | <0.001 | 0.4278 | 1.2961 | 0.938 |
| LysoPC(18:4(6Z,9Z,12Z,15Z)) | 516.3064 | HMDB10389 | <0.001 | 0.3310 | 1.2171 | 0.922 |
| PA(20:4(5Z,8Z,11Z,14Z)e/2:0) | 487.2797 | HMDB11156 | <0.001 | 0.4940 | 1.0228 | 0.900 |
| Lactosylceramide (d18:1/12:0) | 788.5508 | HMDB04866 | <0.001 | 5.0774 | 2.5805 | 0.864 |
| L-Acetylcarnitine | 204.1230 | HMDB00201 | <0.001 | 0.1213 | 2.2395 | 1.000 |
| Palmitic acid | 274.2743 | HMDB00220 | <0.001 | 3.5435 | 2.0363 | 0.889 |
| L-Phenylalanine | 166.0861 | HMDB00159 | <0.001 | 0.1470 | 1.9604 | 1.000 |
| Phytosphingosine | 318.3003 | HMDB04610 | <0.001 | 7.1183 | 1.6272 | 0.965 |
| L-Carnitine | 162.1125 | HMDB00062 | <0.001 | 0.2284 | 1.4370 | 0.995 |
| 4-Hydroxycinnamic acid | 182.0811 | HMDB02035 | <0.001 | 0.3341 | 1.0730 | 0.986 |
| Kinetin | 431.1666 | HMDB12245 | <0.001 | 0.2480 | 1.0199 | 1.000 |
| Bilirubin | 585.2709 | HMDB00054 | <0.001 | 0.0025 | 2.8115 | 0.923 |
| Oleic acid | 281.2482 | HMDB00207 | <0.001 | 0.2079 | 6.4139 | 0.987 |
| Linoleic acid | 279.2326 | HMDB00673 | <0.001 | 0.2231 | 5.7745 | 0.975 |
| Stearic acid | 283.2637 | HMDB00827 | <0.001 | 0.2459 | 5.6303 | 0.998 |
| Docosahexaenoic acid | 327.2324 | HMDB02183 | <0.001 | 0.2160 | 4.5147 | 0.981 |
| Dehydroepiandrosterone sulfate | 367.1580 | HMDB01032 | <0.001 | 0.1431 | 4.4525 | 0.876 |
| Ganglioside GM3 (d18:1/18:1(11Z)) | 1159.7140 | HMDB11929 | <0.001 | 0.3657 | 3.5346 | 0.907 |
| Sphingosine 1-phosphate | 380.2563 | HMDB00277 | <0.001 | 0.0896 | 1.0332 | 1.000 |
| L-Tryptophan | 203.0817 | HMDB00929 | <0.001 | 0.1491 | 1.6581 | 1.000 |
| Docosapentaenoic acid | 329.2479 | HMDB01976 | <0.001 | 0.2632 | 1.1883 | 0.936 |
| PI(18:0/20:4(5Z,8Z,11Z,14Z)) | 885.5480 | HMDB09815 | <0.001 | 0.1812 | 14.3725 | 1.000 |
| LysoPC(17:0) | 508.3404 | HMDB12108 | <0.001 | 0.2755 | 9.0228 | 0.999 |
| PI(18:0/18:2(9Z,12Z)) | 861.5499 | HMDB09809 | <0.001 | 0.3054 | 2.9756 | 1.000 |
| PI(18:1(11Z)/18:3(6Z,9Z,12Z)) | 857.5196 | HMDB09827 | <0.001 | 0.1394 | 2.9647 | 1.000 |
| PI(18:1(11Z)/20:4(8Z,11Z,14Z,17Z)) | 883.5344 | HMDB09833 | <0.001 | 0.2377 | 2.6574 | 0.997 |
| LysoPC(20:4(8Z,11Z,14Z,17Z)) | 1131.6632 | HMDB10396 | <0.001 | 0.3719 | 2.1278 | 0.893 |
| PC(15:0/20:2(11Z,14Z)) | 770.5685 | HMDB07946 | <0.001 | 0.2127 | 2.0549 | 1.000 |
| PI(18:0/22:6(4Z,7Z,10Z,13Z,16Z,19Z)) | 909.5456 | HMDB09821 | <0.001 | 0.3139 | 1.6474 | 1.000 |
| Androsterone sulfate | 369.1736 | HMDB02759 | <0.001 | 0.2075 | 3.1326 | 0.862 |
| Tetrahydrocorticosterone | 349.2375 | HMDB00268 | <0.001 | 0.4176 | 1.1879 | 0.965 |
| Cholesterol sulfate | 465.3040 | HMDB00653 | <0.001 | 0.2506 | 8.6063 | 0.988 |

Table S8 Pathway analysis by MetPA online tool

|  | Total | Expected | Hits | Raw p | -Log(P) | Holm adjust | FDR | Impact |
| --- | --- | --- | --- | --- | --- | --- | --- | --- |
| Biosynthesis of unsaturated fatty acids | 36 | 0.55742 | 5 | 0.000157 | 8.7593 | 0.013188 | 0.013188 | 0 |
| Linoleic acid metabolism | 5 | 0.077419 | 2 | 0.002234 | 6.1038 | 0.18546 | 0.093845 | 1 |
| Sphingolipid metabolism | 21 | 0.32516 | 3 | 0.003616 | 5.6225 | 0.29649 | 0.10124 | 0.0284 |
| Phenylalanine, tyrosine and tryptophan biosynthesis | 4 | 0.061935 | 1 | 0.060569 | 2.804 | 1 | 1 | 0.5 |
| Glycerophospholipid metabolism | 36 | 0.55742 | 2 | 0.10522 | 2.2517 | 1 | 1 | 0.11182 |
| Steroid hormone biosynthesis | 85 | 1.3161 | 3 | 0.14064 | 1.9615 | 1 | 1 | 0.00013 |
| Phenylalanine metabolism | 10 | 0.15484 | 1 | 0.14488 | 1.9319 | 1 | 1 | 0.35714 |
| Aminoacyl-tRNA biosynthesis | 48 | 0.74323 | 2 | 0.16867 | 1.7798 | 1 | 1 | 0 |
| alpha-Linolenic acid metabolism | 13 | 0.20129 | 1 | 0.18426 | 1.6914 | 1 | 1 | 0 |
| Citrate cycle (TCA cycle) | 20 | 0.30968 | 1 | 0.26951 | 1.3111 | 1 | 1 | 0.09038 |
| Alanine, aspartate and glutamate metabolism | 28 | 0.43355 | 1 | 0.35649 | 1.0314 | 1 | 1 | 0 |
| Porphyrin and chlorophyll metabolism | 30 | 0.46452 | 1 | 0.37663 | 0.97648 | 1 | 1 | 0.05288 |
| Glyoxylate and dicarboxylate metabolism | 32 | 0.49548 | 1 | 0.39617 | 0.92591 | 1 | 1 | 0.03175 |
| Arachidonic acid metabolism | 36 | 0.55742 | 1 | 0.4335 | 0.83587 | 1 | 1 | 0 |
| Fatty acid elongation | 39 | 0.60387 | 1 | 0.46003 | 0.77646 | 1 | 1 | 0 |
| Fatty acid degradation | 39 | 0.60387 | 1 | 0.46003 | 0.77646 | 1 | 1 | 0 |
| Tryptophan metabolism | 41 | 0.63484 | 1 | 0.47705 | 0.74013 | 1 | 1 | 0.14305 |
| Fatty acid biosynthesis | 47 | 0.72774 | 1 | 0.52509 | 0.64419 | 1 | 1 | 0.01473 |

Table S9. Random forest method was able to correctly classify subjects of 13 COVID-19 plasma samples in both discovery set and validation set.

Discovery set: Random forest accuracy = 100%

N=100,52metabolites

|  | Virus replication phase | Convalescence | class.error |
| --- | --- | --- | --- |
| Virus replication phase | 64 | 0 | 0.0000 |
| Convalescence | 0 | 36 | 0.0000 |

Validation set: Random forest accuracy = 100%

N=13,52metabolites

|  | Virus replication phase | Convalescence | class.error |
| --- | --- | --- | --- |
| Virus replication phase | 8 | 0 | 0.0000 |
| Convalescence | 0 | 5 | 0.0000 |
